# A Protein Domain Co-Occurrence Network Approach for Predicting Protein Function and Inferring Species Phylogeny

**DOI:** 10.1371/journal.pone.0017906

**Published:** 2011-03-24

**Authors:** Zheng Wang, Xue-Cheng Zhang, Mi Ha Le, Dong Xu, Gary Stacey, Jianlin Cheng

**Affiliations:** 1 Department of Computer Science, University of Missouri, Columbia, Missouri, United States of America; 2 Christopher S. Bond Life Science Center, University of Missouri, Columbia, Missouri, United States of America; 3 Division of Plant Science, University of Missouri, Columbia, Missouri, United States of America; 4 Informatics Institute, University of Missouri, Columbia, Missouri, United States of America; Kyushu Institute of Technology, Japan

## Abstract

Protein Domain Co-occurrence Network (DCN) is a biological network that has not been fully-studied. We analyzed the properties of the DCNs of *H. sapiens*, *S. cerevisiae*, *C. elegans*, *D. melanogaster*, and 15 plant genomes. These DCNs have the hallmark features of scale-free networks. We investigated the possibility of using DCNs to predict protein and domain functions. Based on our experiment conducted on 66 randomly selected proteins, the best of top 3 predictions made by our DCN-based aggregated neighbor-counting method achieved a semantic similarity score of 0.81 to the actual Gene Ontology terms of the proteins. Moreover, the top 3 predictions using neighbor-counting, χ^2^, and a SVM-based method achieved an accuracy of 66%, 59%, and 61%, respectively, when used to predict specific Gene Ontology terms of human target domains. These predictions on average had a semantic similarity score of 0.82, 0.80, and 0.79 to the actual Gene Ontology terms, respectively. We also used DCNs to predict whether a domain is an enzyme domain, and our SVM-based and neighbor-inference method correctly classified 79% and 77% of the target domains, respectively. When using DCNs to classify a target domain into one of the six enzyme classes, we found that, as long as there is one EC number available in the neighboring domains, our SVM-based and neighboring-counting method correctly classified 92.4% and 91.9% of the target domains, respectively. Furthermore, we benchmarked the performance of using DCNs to infer species phylogenies on six different combinations of 398 single-chromosome prokaryotic genomes. The phylogenetic tree of 54 prokaryotic taxa generated by our DCNs-alignment-based method achieved a 93.45% similarity score compared to the Bergey's taxonomy. In summary, our studies show that genome-wide DCNs contain rich information that can be effectively used to decipher protein function and reveal the evolutionary relationship among species.

## Introduction

Biological systems, such as living cells, are composed of a large number of individual components (e.g., proteins, DNA, RNA, and small molecules). These molecules interact and form networks to carry out biological functions. Departing from the traditional reductionist approach of studying single targets, systems biology aims to identify the cellular molecular components and their interactions and analyze cellular responses at a large scale using high-throughput experimental techniques (e.g., genome sequencing, DNA microarrays, yeast two-hybrid experiments, proteomics, and metabolomics) and computational methods [1]–[7]. One promising approach to analyze the complex interactions among molecules is *network biology* – studying the structure, dynamics, and function of biological networks at the system level [8].

Currently, network biology primarily focuses on metabolic, gene regulatory, and/or protein-protein interaction networks [9]–[20]. Since proteins and their interactions play central roles in almost all biological processes, protein interaction networks have been a major target of network biology. Experimental techniques, such as yeast two-hybrid [21] and many computational methods have been developed to construct protein-protein interaction networks. The network approach to the study of protein interactions has shed light not only on the general principles that govern the evolution and functions of the proteins in a species (i.e. proteome) as a whole, but also the function and roles of a particular protein of interest. For instance, protein interaction networks can be used to identify hub proteins having critical biological functions, to predict biological pathways, and to infer the function of a protein according to its interactions with other proteins with known functions [22]–[24].

Despite many successful applications, the study of protein interaction networks is hindered by two serious problems [14], [25]. First, protein interaction networks constructed from high-throughput experimental techniques, such as yeast two-hybrid (Y2H), have a high level of false positives [14]. It was estimated that more than half of the protein interactions in an Y2H protein interaction network may be false positives [25]. Protein interactions predicted by computational methods are even noisier. Second, current protein interaction networks constructed for most species are far from complete. For instance, estimates suggest that only about 10% of the protein interactions in the human genome have been elucidated to date [14]. Thus, inferring protein function, interaction, and evolution from protein interaction networks might not be accurate and reliable.

Here, we propose to use protein *domain co-occurrence networks* (DCN) [26], [27] to study the function and interaction of proteins at the proteome level. These networks make use of the co-occurrences of various protein domains in given proteins. A protein domain, usually a segment of continuous sequence within a protein, is considered the structural, functional, and evolutionary unit of proteins. One protein is generally composed of one or more domains (i.e. building blocks) that each might fold independently into a stable structure. Each domain often has a distinct biochemical function. Domains that are similar in sequence, structure, and function are grouped together in families/types. The proteome (i.e. the collection of all the proteins) of a species usually has representatives of thousands of domain types. These domains can exist as single-domain proteins or are combined together to form multi-domain proteins. Hence, in addition to sequential divergence, domain combination is another major mechanism of increasing the complexity of a proteome [28]. Nature tends to reuse and recombine existing building blocks to create new proteins, rather than to invent them *de novo* [29].

**Figure 1:**
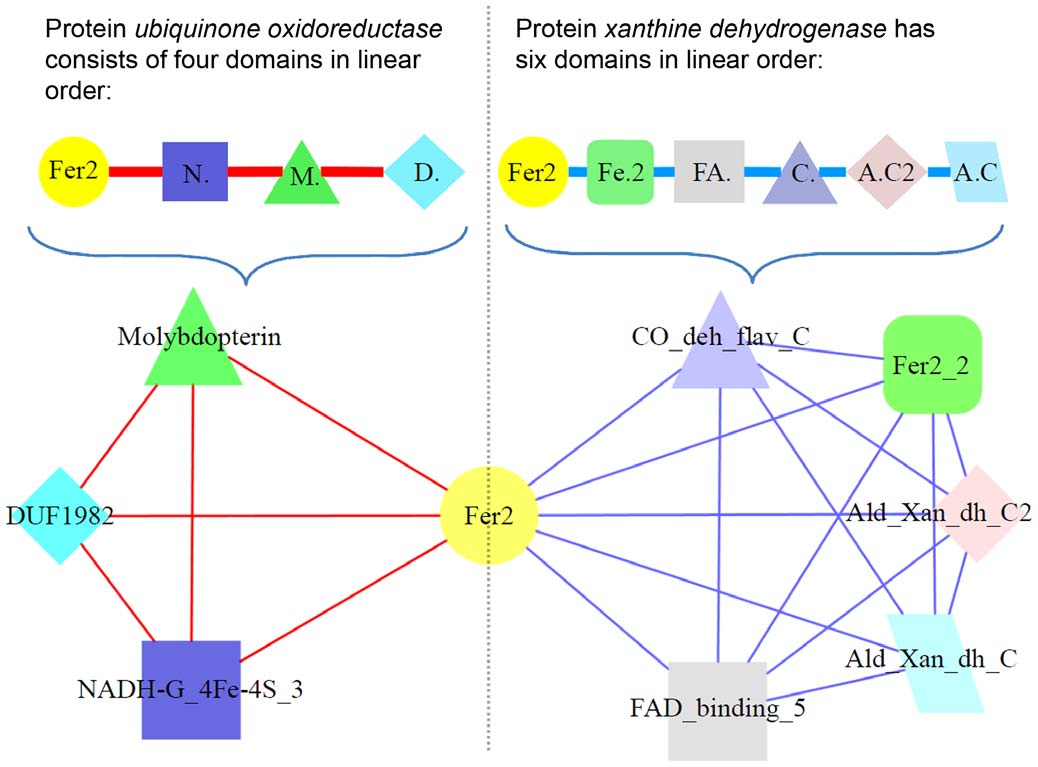
A small DCN consisting of two Arabidopsis proteins. Left: Protein *ubiquinone oxidoreductase*, which contains four Pfam domains. Right: Protein *xanthine dehydrogenase*, which has six Pfam domains. The two proteins share the same domain *Fer2*. An edge is drawn between domains co-occurring in the same protein. A fully connected sub-graph (clique) in the DCN corresponds to a protein.

Domain combination represents a strong, permanent and definite interaction between domains, which can be captured by *domain co-occurrence networks* (DCN). A DCN is a graph consisting of all the protein domain types of a species as nodes. Two domain types (i.e. nodes) are connected by an edge if they co-exist in one protein [26]. **Figure 1** shows the domain architecture (i.e., a series of domain types) of two multi-domain proteins in Arabidopsis and a DCN derived from the two proteins. Previous studies showed that DCNs of the yeast and human were scale-free and small-world networks [26], like other networks such as web hyper-link networks, social networks, and protein interaction networks. However, to date, the features and properties of DCNs have not been well explored, and they have not been used to study the functions and evolution of proteins. Compared to the well-studied protein-protein interaction networks, DCN has the following advantages, making it a very promising tool for studying proteomes at the system level: (1) **Accurate and reliable**. Domain co-existence (or combination) relationship constructed from sequential analysis is almost 100% accurate, which is much more reliable than protein interactions predicted from experimental approaches (e.g., yeast two-hybrid). (2) **Higher coverage**. A DCN constructed from homologous sequence analysis usually can recall about 70% of domain co-existence relationships [30], compared to the very low coverage of protein interaction networks of most species. Thus, the inference based on DCNs is often more reliable. (3) **Easy to construct**. It is much easier to construct the DCN of a genome by comparing its protein sequences against known protein domain databases, such as Pfam [31] and ProDom [32], compared to building protein interaction networks through either experimental or other computational methods.

In addition, DCNs also have two other distinct features. First, each node represents a domain type instead of an instance (i.e. sequence). Since DCNs of differing species share a large number of common domain types, DCNs can be more readily compared or aligned across species than sequence-based networks, such as protein-protein interaction networks. Second, an edge in a DCN represents a permanent combination relationship that is stronger than the relationship defined in protein-protein interaction networks, which is often intangible. Therefore, DCNs can provide richer information about the functional relationship between connected domains. Therefore, DCN is a very valuable target for biological network research and a useful tool for studying the evolution, function, and interaction of proteins.

In this study, we constructed the DCNs for *H. sapiens*, *S. cerevisiae*, *C. elegans*, *D. melanogaster*, and 15 plant genomes and performed statistical analysis and attack simulations. A “domain” used in our study is a Pfam entry, corresponding to a Pfam domain “family”, such as “*Pkinase_Tyr* (PF07714)” and “*Helicase_C* (PF00271)” [33]. The Pfam domains are often more like protein function units than structural ones. We utilized DCNs to predict domain functions, including GO terms or enzyme classes, and inferred prokaryotic species phylogenies for the first time. Our large-scale studies of DCNs on this diverse set of species demonstrate that DCNs can be readily and reliably constructed from a genome or a list of proteins of an organism, and, in conjunction with graph neighboring methods, can be used to effectively predict protein functions and accurately infer species phylogenies.

## Results and Discussion

### Statistical Properties of Domain Co-occurrence Networks

**Figure 2:**
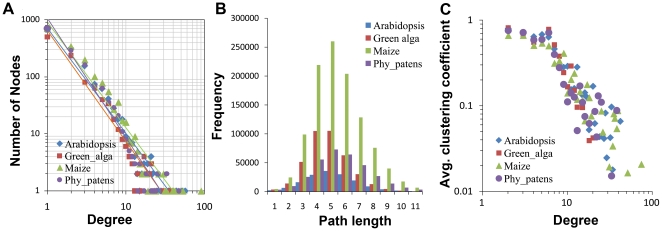
Statistical properties of DCNs of four representative species. (**A**) The degree distribution plots (scale-free); (**B**) the distributions of lengths of the shortest paths (small-world); (**C**) the log-log plots of clustering coefficients against degrees (hierarchical modularity).

We analyzed the statistical properties of the DCNs of 15 plant species, yeast, and human, and found that they share several common features. **Figure 2** (A) depicts the node-degree distribution of four example species, *Arabidopsis thaliana* (Arabidopsis), *Chlamydomonas reinhardtii* (green alga), *Zea mays* (maize), and *Physcomitrella patens* (moss). Supplementary **Figure S1** shows the node-degree distributions of *H. sapiens*, *S. cerevisiae*, *C. elegans*, *D. melanogaster*, and 15 plant genomes. This log-log plot shows that the number of nodes with a specific degree value (degree is the number of edges linked to a node) mathematically follows a power law distribution, because the logarithmic relationship between two variables approximates a linear relationship. This property shows that the DCNs are scale-free networks
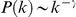
where *P*(*k*) is the probability of having a node with *k* edges linking to it, and γ is a species-specific constant.

**Figure 2 **(**B**) plots another property of scale-free networks, the small-word phenomenon. **Figure 2 **(**B**) shows the frequency of node pairs whose shortest path has a specific value *k* (*k* = *1, 2, 3*…). Supplementary **Figure S2** shows the shortest path length distributions of *H. sapiens*, *S. cerevisiae*, *C. elegans*, *D. melanogaster*, and 15 plant genomes. The majority of the node pairs have a path shorter than *5*, indicating that most of node pairs can be reached within five steps.

Average clustering coefficient is another important property of scale-free networks. For un-directed networks, the clustering coefficient of a node *n* is calculated by:
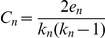
Where *e_n_* is the number of connected node pairs among immediate (one edge away) neighboring nodes of the central node *n*, and *k_n_* is the number of immediate neighboring nodes of the central node *n* [8], [34]. Clustering coefficient indicates the degree of the nodes to be clustered. The average clustering coefficient of the whole network is calculated by taking the average of all nodes' clustering coefficients. **Figure 2 **(**C**) plots the relationship between average clustering coefficient and node degrees. This log-log plot shows that the clustering coefficient distribution of DCNs also follows a power law. Supplementary **Figure S3** shows the average clustering coefficient distributions of *H. sapiens*, *S. cerevisiae*, *C. elegans*, *D. melanogaster*, and 15 plant genomes. **Figure 2 **(**B**) and (**C**) illustrate that in DCNs, the nodes with a small degree value, usually tend to form a complete graph (clique), or very dense sub-graph (i.e. with higher average clustering coefficient), and one dense sub-graph is connected with other dense sub-graphs by hub nodes, i.e. popular nodes with high degree, which are similar to politicians and celebrities in human social networks. This indicates that a DCN is a hierarchical network composed of densely connected modules [35].

Experiments conducted on yeast and human DCNs show that they follow the same properties (data not shown). Therefore, they share the same characteristics as other scale-free networks, such as social networks, the World-Wide Web, and some biological networks, including protein-protein interaction networks and metabolic networks.

### Error and attack robustness of DCNs

Scale-free networks are usually very recalcitrant (remains un-fragmented, i.e., every node is connected, by some paths, to the others) to random removal of nodes, named as “failure”, but very vulnerable to “attack”, in which the nodes with highest degree are removed first [36]–[38]. We simulated both perturbations on DCNs.

The Average Shortest Path (ASP) of a network is the average of all the shortest paths between all pairs of nodes. A smaller ASP means the network has a better interconnectivity. If a network is fragmented, i.e., containing several independent sub-graph(s) whose nodes have no connections to the other sub-graphs, the path between these un-connected nodes become infinity, so does the ASP of the entire networks. As mentioned in the “**Materials and Methods**”, the DCN for a given species usually contains several disconnected sub-graphs, with one main graph containing approximately >80% of all the domains. Therefore, it only makes sense to study the changes of ASP under the two perturbations (“failure” and “attack”) on the main network. According to our criteria, the simulations were terminated and the network was considered fragmented even if one node became fragmented. This is probably a too stringent criteria compared with the study performed on yeast protein-protein interaction networks [36], where the shortest path between disconnected nodes were treated a large number instead of infinity. That criteria allows calculating the interconnectivity of a network after some nodes are fragmented.

We performed simulations on the DCNs of yeast, Arabidopsis, maize, soybean, and human. Taking the main network of Arabidopsis as an example, after removing the node with the highest degree, domain *Helicase_C*, the ASP of the network went to infinity, indicating that this attack behavior caused at least one node to be disconnected with the remaining network (i.e. network fragmented). Compared with “attack”, scale-free networks are usually less vulnerable to “failure”, in which nodes are removed randomly, because the probability of randomly selecting a node with high degree value is very low, according to the power law distribution. This trend is also found true in DCNs, but to a lesser extent. We performed 10 rounds of failure simulations on the DCNs of human, yeast, and Arabidopsis, and the average number of nodes deleted before the DCN had at least one nodes fragmented were 5.5, 4.0, and 7.3, with standard deviations 4.2, 3.5, and 5.9, respectively. These are the 0.4%, 1.6%, and 1.6% of all the vertices in their main networks, respectively.

The DCN was considered fragmented even if one node became fragmented. In order to study the interconnectivity of the networks after some of the nodes became fragmented and to make comparison with the study performed on yeast protein-protein interaction networks [36], instead of using infinity, we assigned a specific value as the shortest path when two nodes were disconnected. The value was equal to the maximum length of the shortest paths before the removal action. We observed that DCNs were very vulnerable to “attack”, i.e., the modified ASP had a sharp increase and reached the peak after removing 2.5% and 5% of the nodes of yeast and rice, respectively, and much more robust against “failure”, i.e., the modified ASP had a much slower increase compared to “attack”. However, we found that DCNs were not as robust as yeast interaction networks under “failure”, as the ASP in yeast interaction networks remained almost unchanged after removing up to 50% of nodes based on study [36].

In summary, the results show that DCNs follow the general robustness characteristics of scale-free networks, but are more vulnerable to perturbations than protein interaction networks and other high-density scale-free networks. One possible reason is that the “domain” used in our DCNs sometime may be a coarse unit, which sometime may be further divided into smaller units. The other reason is probably because DCNs are less densely connected than protein interaction networks and the internet, or because of the much smaller size of DCNs. This may indicate that the interconnectivity of DCNs is not indispensably important, mainly because of the nature of DCNs. For the networks showing great robustness, their functions can only be carried out by interconnectivity, such as the Internet. On the other hand, for the domains that do not have a direct connection in the DCN, they may also be able to physically interact with the others and carry out functions. For example, two domains from two different proteins may be interactive and bind with each other, but they do not co-exist in one protein.

### Domain function prediction – GO terms

To assess the capability of DCN to predict the GO terms of a target domain, we developed and evaluated three methods: neighbor-counting, χ^2^, and a SVM-based method, as shown in **Table 1**. The neighbor-counting method retrieves the GO terms of all neighboring vertices and ranks them by their occurrence frequencies. This ranked list of GO terms is its final predictions. The χ^2^ method not only considers the occurrence frequencies of GO terms in the neighboring nodes, but also the overall distributions of the GO terms in the entire DCN. The SVM-base method uses the known examples of the target domains to train a SVM model, and then uses this SVM model to make predictions. Details can be found in the “Materials and Methods” section. We evaluated the top 3 ranked GO terms by the criteria that if one of the top 3 GO terms matches one of the real GO terms of the target domains, we count it as a correct prediction. Similarly, the top 1 GO term evaluation criteria is that only if the top 1 GO term matches one of the real GO terms, it is considered as a correct prediction. For the neighbor-counting and χ^2^ value methods, we calculated the percentage of correctly predicted domains. For the SVM-based method, we performed a leave-one-domain-out cross-validation, and report the average percentage of correctly predicted domains. These three methods were tested on the target domains whose “radius one” neighboring domains have at least one GO term available. The number of target domains for Arabidopsis, yeast, and human are 736, 518, and 953, respectively.

**Table 1:** The prediction accuracy of using neighbor counting, χ2, and SVM when predicting GO terms.

Species	Top 3			Top 1		
	Neigh.-Count.	χ^2^	SVM	Neigh.-Count.	χ^2^	SVM
Arabidopsis	64.8%	59.6%	58.5%	50.4%	42.9%	47.6%
Yeast	67.0%	61.8%	61.1%	52.3%	41.9%	51.6%
Human	65.8%	58.3%	60.4%	47.4%	37.5%	45.1%

For SVM, it's the average accuracy of a leave-one-domain-out cross-validation. “Top 1” (“Top 3”) indicates when “the top one ranked GO term” (“one of the top three ranked GO terms”) matches one of the actual GO terms of the target domain, it is considered a correct prediction. The values shown under “Top 1” are also the precision values for top 1 prediction. The precision values for “Top 3” predictions can be found at **Table 4**.

The evaluation criteria mentioned above is a yes-or-no binary criteria. Considering that two different GO terms may share functional similarity, we also calculated the average similarity score between the best prediction and the real GO terms (**Table 2**), using the tool G-SESAME [39], which defines the semantic similarity between two GO terms as the percentage of their common sub-graphs starting from the root of the GO “directed acyclic graph” (DAG) [39].

**Table 2 pone-0017906-t002:** The average semantic similarity scores of the best predictions, among “Top 1” or “Top 3” GO term(s).

Species	Top 3			Top 1		
	Neigh.-Count.	χ^2^	SVM	Neigh.-Count.	χ^2^	SVM
Arabidopsis	0.814	0.790	0.767	0.680	0.652	0.620
Yeast	0.835	0.818	0.772	0.679	0.646	0.626
Human	0.826	0.790	0.791	0.665	0.619	0.630

“Top 1” (“Top 3”) indicates that for each target domain, we calculate the pair-wise similarity scores between the top one (top three) ranked GO term(s) and the actual GO terms, and the highest score is considered as the similarity score of the best prediction. Semantic scores are calculated by the tool G-SESAME [39].

For the SVM-based method, we tested each of the five set features built on GO terms, EC numbers, amino acid sequences, secondary structures, and solvent accessibilities. We first used only GO term frequency and kept adding one of the other features. After comparing the performances of several leave-one-domain-out cross-validations, we found that the “GO terms frequency” feature, generated from the neighboring domains, had the largest positive influence on final prediction accuracy. Adding any one of the remaining features slightly decreased the accuracy by 1–3% on Arabidopsis. We also tested four kernel functions for the SVM model: linear, polynomial, radial basis function (RBF), and sigmoid tanh, and found that the linear kernel function yielded the best accuracy. Therefore, the SVM method used in our evaluations (**Tables 1** **and** **2**) only uses “GO term frequency” features consisting of 31,398 values and the linear kernel function. Moreover, we randomly selected different numbers of negative examples in each GO term's training dataset (see “**Materials and Methods**” section for how to construct a training dataset), and found that the ratio 2∶1 negative to positive examples generates the best accuracy, which is reported in **Tables 1** **and** **2**.

**Figure 3:**
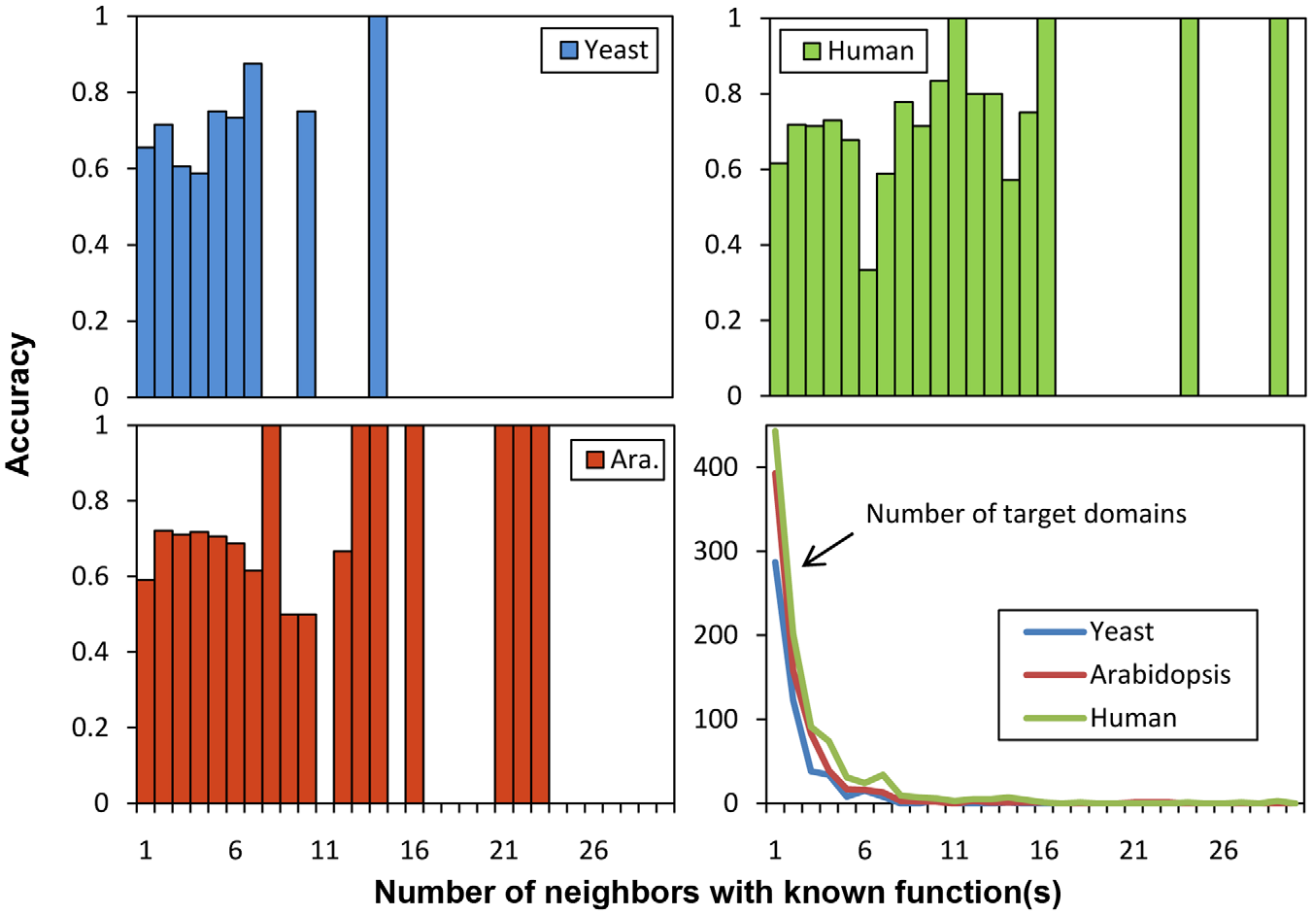
The number of neighbors with known functions versus the prediction accuracy of the neighbor-counting method. The more neighors with known functions, the higher accuracies neighor-counting method can achieve when pedicting the functions of the central node. Most of the target domains have less than five neigbors with known functions.

Our results (**Table 1** **–** [Table pone-0017906-t002] [Table pone-0017906-t003] **4**) show that the SVM-based method has the better performance compared to the χ^2^-based method when considering only top 1 predictions; and the simple neighbor-counting method, which relies solely on DCN topology, achieves the best performance, in terms of both the “yes-or-no” evaluation and the average similarity between the best predictions and real GO terms. The accuracy of top 3 predictions using neighbor counting on three species is more than 65% and top 1 predictions more than 47%. The average GO similarity of top 3 predictions is more than 0.81 and top 1 prediction more than 0.66. The good performance suggests that the connectivity of DCNs contains rather rich information for protein function prediction. To further investigate the neighbor counting method, we plotted **Figure 3**, the relationship between “the number of neighboring domains with known function” and “the prediction accuracy” (top 3, radius one neighboring domains) on three test species: Arabidopsis, yeast, and human. The results showed that, in general, more neighboring domains with known functions can generate better accuracy. Although most of the target domains have less than five neighboring domains with known function, as long as there is one neighboring domain with functional information available, neighbor-counting can correctly predict 60% of the domains (top 3). This shows that domain co-occurrence is a very useful indicator of protein function.

**Table 3 pone-0017906-t003:** The average recall value of using neighbor counting, χ2, and SVM when predicting GO terms.

Species	Top 3			Top 1		
	Neigh.-Count.	χ^2^	SVM	Neigh.-Count.	χ^2^	SVM
Arabidopsis	47.0%	42.5%	40.4%	21.4%	18.6%	20.0%
Yeast	47.4%	44.3%	41.5%	22.0%	18.0%	20.9%
Human	45.8%	40.1%	40.4%	19.8%	16.5%	19.1%

Recall was calculated as the correctly predicted GO terms divided by the total number of real GO terms. Average recall was the sum of recall value for each domain divided by the total number of test domains.

**Table 4:** The average precision value of using neighbor counting, χ2, and SVM for top 3 predictions.

Species	Top 3		
	Neigh.-Count.	χ^2^	SVM
Arabidopsis	40.7%	36.8%	35.3%
Yeast	41.3%	38.0%	36.9%
Human	38.9%	34.1%	34.7%

Precision was calculated as the number of correctly predicted GO terms divided by the number of predictions a method made (in this case, three). Average precision was the sum of precision value for each domain divided by the total number of test domains. The precisions of “Top 1” can be found at **Table 1**.

We also conducted experiments on integrating both radius one and two neighboring domains. For neighbor-counting and SVM-based method, our results show that the accuracy has a small decrease, by 2–3% on average, after including radius two neighbors (data not shown). For the χ^2^-based method, the accuracy drops by 10% on average (data not shown). This indicates that directly expanding the neighboring radius may not help improving accuracy, as more noise may be included. In the future, we plan to incorporate more advanced algorithms such as graph kernels [40] and graph random walk algorithm [41] to infer functions from DCNs.

We selected nine most promiscuous domains [42] (i.e., the hub domains or the vertices in DCNs with a high degree) and used the three methods: neighbor-counting, χ^2^, and SVM-based method, to predict their functions (**Table 5** **–** [Table pone-0017906-t006] **7**). Our results show that in general, if considering top 3 hits, the neighbor-counting method performs better than χ^2^ and SVM-based method. However, SVM-based method performs better on Arabidopsis and human if considering only top 1 prediction. Moreover, for some promiscuous domains, χ^2^ and SVM-based method perform better than neighbor-counting. For example, the top 1 prediction of neighbor-counting method has a similarity score of 0.088 on human domain “*Pkinase_Tyr*”, whereas χ^2^ method has a score of 1.000 on the same domain. Similarly, the best prediction of neighbor-counting has a similarity score of 0.080 on human domain “*SET* ”, whereas the SVM-based method has a score of 1.000. This shows that each of these three methods may have its own advantages when being applied to specific domains. **Table 5** also shows that usually the higher degree value a domain has, the better performance neighbor-counting method can achieve if considering top 3 predictions.

**Table 5:** Performances of neighbor-counting method on promiscuous domains.

Domain	Degree			Top 3			Top 1		
	Arab.	Yeast	Hum.	Arab.	Yeast	Hum.	Arab.	Yeast	Hum.
Helicase_C	44	24	55	1.000	1.000	1.000	0.387	1.000	1.000
PDZ	1	N/A	69	0.234	N/A	1.000	0.115	N/A	0.160
Pkinase	44	15	63	1.000	1.000	1.000	1.000	1.000	0.300
Pkinase_Tyr	17	N/A	41	1.000	N/A	1.000	0.246	N/A	0.088
Pkinase_C	1	4	14	1.000	1.000	1.000	1.000	0.171	0.300
PHD	35	11	43	1.000	0.557	1.000	0.096	0.557	0.557
AAA	24	21	21	1.000	1.000	1.000	1.000	1.000	1.000
SET	12	2	20	1.000	0.096	1.000	0.054	0.096	0.080
GATase	7	13	11	0.538	0.262	0.602	0.538	0.220	0.602
Average	20.6	12.9	37.4	0.864	0.702	0.956	0.493	0.578	0.454

Definition of best prediction can be found at the caption of **Table 2**. “N/A” indicates this specific domain does not exist in the DCN of a species. “Average” indicates the average value of degree values and similarity scores. “Arab.” indicates “Arabidopsis”, and “Hum.” indicates “Human”. Some of these promiscuous domains were selected from [42].

**Table 6 pone-0017906-t006:** Performances of χ2 method on promiscuous domains.

Domain	Top 3			Top 1		
	Arabidopsis	Yeast	Human	Arabidopsis	Yeast	Human.
Helicase_C	1.000	0.696	1.000	1.000	0.696	0.696
PDZ	0.234	N/A	0.482	0.234	N/A	0.284
Pkinase	1.000	1.000	1.000	1.000	1.000	0.573
Pkinase_Tyr	0.670	N/A	1.000	0.670	N/A	1.000
Pkinase_C	1.000	1.000	0.206	0.895	0.895	0.199
PHD	0.669	0.557	0.661	0.000	0.557	0.557
AAA	0.152	0.152	0.152	0.040	0.040	0.040
SET	0.058	0.096	0.488	0.058	0.096	0.058
GATase	0.302	0.602	0.602	0.538	0.602	0.602
Average	0.560	0.584	0.617	0.486	0.552	0.442

Definition of the best prediction can be found at the caption of **Table 2**. The degree value of each promiscuous domain can be found at **Table 5**.

**Table 7:** Performances of SVM-based method on promiscuous domains.

Domain	Top 3			Top 1		
	Arabidopsis	Yeast	Human	Arabidopsis	Yeast	Human.
Helicase_C	1.000	1.000	1.000	1.000	0.527	1.000
PDZ	0.000	N/A	0.226	0.120	N/A	0.226
Pkinase	0.202	0.171	0.181	1.000	0.037	0.173
Pkinase_Tyr	0.662	N/A	0.132	0.662	N/A	0.088
Pkinase_C	1.000	1.000	1.000	1.000	0.171	0.149
PHD	1.000	0.481	0.557	0.096	0.481	0.096
AAA	1.000	0.081	1.000	1.000	0.039	1.000
SET	1.000	0.485	1.000	1.000	0.000	1.000
GATase	0.337	0.527	0.602	0.079	0.527	0.602
Average	0.689	0.535	0.633	0.662	0.255	0.482

Definition of the best prediction can be found at the caption of Table 2. The degree value of each domain can be found at Table 5.

### Domain function prediction – Enzyme

For a target domain, we first used SVM and a neighbor-inference method to predict whether it is an enzyme domain. On enzyme domains, we tested two methods, neighbor-counting and SVM-based method, to classify it into one of the six enzyme classes.

For SVM-based enzyme “yes or no” predictions, we found that using “GO term frequency” feature with linear kernel function generated the best accuracy. Adding other features such as EC number, secondary structure, and solvent accessibilities did not significantly influence performance (data not shown). **Table 8** **and** **9** compare the performance of using SVM and another method: neighbor-inference, by which if the neighboring domains have at least one EC number, it is predicted as an enzyme domain; otherwise, not. We calculated and report the sensitivity of positives (Q_p_), the sensitivity of negatives (Q_n_), and the Matthews correlation coefficient (MCC) [43]:
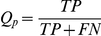


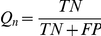


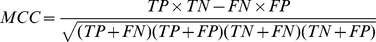
where TP is true positive, FN is false negative, TN is true negative, and FP is false positive. The *Q _p_* and *Q_ n_* of SVM-based method are 0.66 and 0.87 on average, which indicates the predictions are more biased towards the negative side. However, the neighbor-reference method shows more balanced results, with the *Q _p_* and *Q _n_* of 0.79 and 0.78. The overall classification accuracy and the Matthews correlation coefficients of these two methods are very similar (i.e., about 0.54).

**Table 8:** The leave-one-out cross-validation results of the SVM-based enzyme “yes or no” predictions.

Species	Positive	Negative	*TP*	*FN*	*TN*	*FP*	*Q_p_*	*Q_n_*	*MCC*	Ratio
Arabidopsis	309	432	205	104	374	104	0.66	0.87	0.54	0.78
Yeast	220	300	40	75	260	40	0.66	0.87	0.54	0.78
Human	312	646	185	127	593	53	0.59	0.91	0.55	0.81

“Ratio” standards for the percentage of correctly predicted domains in the cross-validation. The feature used are only GO term frequencies gained from radius one neighboring domains.

**Table 9:** The accuracies of using “neighbor-inference” method for enzyme “yes or no” predictions.

Species	Positive	Negative	*TP*	*FN*	*TN*	*FP*	*Q_p_*	*Q_n_*	*MCC*	Ratio
Arabidopsis	309	432	240	69	328	104	0.78	0.76	0.53	0.77
Yeast	220	300	177	43	231	69	0.80	0.77	0.56	0.78
Human	312	646	242	70	497	149	0.78	0.77	0.52	0.77

The “Ratio” indicates the percentage of correctly predicted domains.

To classify an enzyme domain into one of six enzyme classes, we developed and evaluated two methods, neighbor-counting and a SVM-based method (**Table 10**). For the neighbor-counting method, the most frequent enzyme class in the neighboring domains is considered as the predicted enzyme class for the central node. For the SVM-based method, we tested several combinations of the five occurrence-frequency features built on GO terms, EC numbers, amino acid sequences, secondary structures, and solvent accessibilities, and found that using “GO term frequencies” plus “EC number frequencies” generated the best performance in the leave-one-domain-out cross-validation. Adding any one of the other features slightly decreased or did not influence accuracy. As shown in **Table 10**, when there are > = 2 EC numbers available in the neighboring domains, both SVM and neighbor-counting method (top 3) can achieve 100% classification accuracy. Neighbor-counting cannot work on the cases of 70 target domains whose neighboring nodes do not have an EC number, which results in the decrease of its accuracy to 0.711. This is a case on which the neighbor-counting method cannot work, but SVM can make predictions based on the “GO term frequencies” features. In general, as long as there is one EC number in the neighboring domains, the accuracy of both SVM-based and neighboring-counting can reach >90%. This demonstrates the abilities of DCNs to infer enzyme classes. In the future, we will test the performances of inferring sub-classes of enzyme domains.

**Table 10:** Prediction accuracy of the neighbor-counting and SVM-base method when predicting EC families.

Known EC	SVM	Neighbor-Counting		Target Domain Number
		Top 3	Top 1	
> = 0	0.803	0.711	0.674	307
> = 1	0.924	0.924	0.919	237
> = 2	1.000	1.000	0.989	89
> = 3	1.000	1.000	1.000	43
> = 4	1.000	1.000	1.000	21
> = 5	1.000	1.000	1.000	19
> = 6	1.000	1.000	1.000	17
> = 7	1.000	1.000	1.000	6

For SVM, it reports the accuracy of a leave-one-domain-out cross-validation. Experiments were performed on Arabidopsis DCNs. “Known EC” indicates the number of known EC numbers occurrences in the radius one neighboring domains. “Target Number” indicates the number of target domains. The features used in SVM-based method are the GO and EC number occurrence-frequencies, with the linear kernel function.

We also tested other kernel functions in the SVM model and tried to integrate radius two neighboring domains. Linear kernel function worked the best, and integrating more neighboring domains slightly decreased the accuracy (data not shown).

### Protein function prediction

In order to test the performance of applying the aggregated neighbor-counting method to predict protein functions in a real life scenario, we randomly selected 100 proteins from the Gene Ontology FASTA sequence database (http://archive.geneontology.org/lite/2010-12-11/go_20101211-seqdb-data.gz). The functions of these 100 proteins had been annotated, and their GO terms stored in the Gene Ontology database. Therefore, we treated these GO terms as their real GO terms. We ran HHsearch [44] to search each query sequence against the Pfam profile database to detect Pfam domains. Only the Pfam domains detected with an e-value< = 0.01 were kept. To determine the closest relevant species for a query sequence, we used PSI-BLAST [45] to search each of the 100 proteins against the whole genome protein sequences of *H. sapiens*, *S. cerevisiae*, *C. elegans*, *D. melanogaster*, 15 plants species, and 398 single-chromosome prokaryotic species. In total, 96 proteins had at least one PSI-BLAST hit with an e-value< = 0.01, and the other four had an e-value: 1.8, 1.9, 2.7, and 3.7, respectively. The DCN of the species of the most significant hit of a protein is used to predict is function.

In total, DCN-based aggregated neighbor-counting method (details see “Materials and Methods” section) generated predictions for 66 out of 100 proteins. Among the 34 proteins on which DCNs failed to make predictions, 10 of them failed because no Pfam domain was detected by HHsearch with an e-value< = 0.01; 19 of them failed because the Pfam domain(s) detected by HHsearch could not be found in the DCN of the closest relevant species; and the others failed because none of their neighboring domains had GO terms available. Because we used PfamScan, a sequence-profile alignment tool, to detect Pfam domains when we constructed the DCNs (see the “Construction of Domain Co-occurrence Networks” sub-section in the “Materials and Methods” section) but used HHsearch, a much more sensitive profile-profile alignment tool, to detect Pfam domains on these 100 proteins, some of the domains found by HHsearch could not be found by PfamScan. This also suggests that the coverage of our current DCNs can be improved by using more sensitive domain detection tools. Another reason why some of the detected domains cannot be found in the DCNs is that we used PSI-BLAST to identify relevant species, which may not be the right species for the query protein. Thus, adding the DCNs of more species into our system may increase the coverage of function prediction.

**Figure 4:**
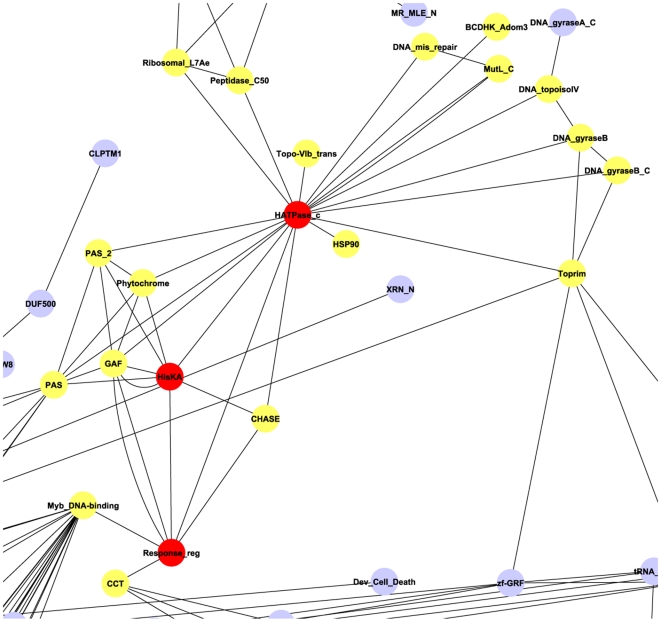
An example showing the DCN-based aggregated neighbor-counting method for protein function prediction. The query protein for this example contains 1,225 amino acids and has an ID “DDB_G0274191” in DictyBase (http://dictybase.org/gene/DDB_G0274191). Three unique Pfam domains (red vertices) were detected by HHsearch, each with an e-value< = 0.01. It had a PSI-BLAST hit to a protein of *Vitis vinifera* (grape) with an e-value e-118. Therefore, the DCN of *Vitis vinifera* was used to make predictions. The vertices in yellow are the radius one neighboring vertices of the three Pfam domains. The GO terms of these yellow vertices were put together and ranked based on their occurrence frequencies. This query protein contains 14 real GO terms. Everyone of the top 3 predictions (GO:0005524, GO:0006355, and GO:0016020) ranked by DCN-based aggregated neighbor-counting method matches one of the real GO terms. Besides that, the 5th (GO:0007165), 7th (GO:0000155), 13th (GO:0000160), and 14th (GO:0000156) ranked GO terms have an exact match to the real GO terms.

**Figure 5:**
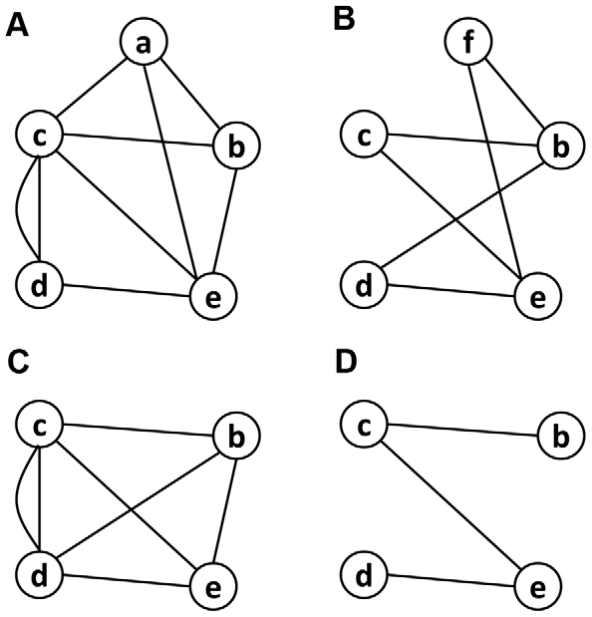
An example illustrating the graph alignment algorithm we utilized.

**Table 11** reports the average precision, recall, and the semantic similarity score of the best predictions on the 66 proteins including both multi- and single-domain proteins. **Table 12** shows the same measurements on the 9 single-domain proteins containing only one Pfam domain (i.e., only one domain with an HHsearch e-value< = 0.01). Precision was calculated as the number of correctly predicted GO terms (“exact match”) divided by the number of GO predictions. Recall was calculated as the correctly predicted GO terms (“exact match”) divided by the total number of real GO terms. Our results show that the top 1 prediction on single-domain proteins can achieve a similarity score of 0.636. Considering top 3 predictions on single-domain proteins, the best prediction has a similarity score of 0.834, and above 0.95 if considering more than top 4 predictions. **Figure 4** shows a successful prediction example for one protein, in which all top 3 predictions match the real GO terms. For both domain function and protein function predictions, if more than two edges existed between two vertices, as the case shown in **Figure 5** between node *c* and *d*, they were reduced to one edge.

**Table 11:** The results of DCN-based aggregated neighbor-counting method on 66 randomly selected proteins.

Number of top predictions	Average Precision	Average Recall	Average similarity score of best prediction
1	36.4%	6.5%	0.600
2	33.3%	11.5%	0.724
3	30.3%	18.8%	0.805
4	26.9%	21.9%	0.855
5	23.9%	23.6%	0.874
6	23.2%	27.8%	0.897
7	21.2%	29.2%	0.913
8	19.9%	31.1%	0.913
9	18.6%	32.1%	0.913
10	17.1%	32.8%	0.913

The ways of calculating precision and recall can be found at the captions of **Table 4** and **Table 3**, respectively. Explanations of the best semantic similarity score can be found at the caption of **Table 2**. From the 100 proteins randomly selected from GO database, 66 proteins have predictions available by DCN-based aggregated neighbor-counting method.

**Table 12:** The results of DCN-based aggregated neighbor-counting method on 9 single-domain proteins.

Number of top predictions	Average Precision	Average Recall	Average similarity score of best prediction
1	33.3%	8.4%	0.636
2	27.8%	14.3%	0.799
3	22.2%	15.6%	0.834
4	27.8%	21.6%	0.960
5	22.2%	21.6%	0.960
6	20.4%	25.4%	0.960
7	17.5%	25.4%	0.960
8	16.7%	27.6%	0.960
9	16.0%	31.3%	0.960
10	15.6%	32.2%	0.960

### Evaluation of DCN-Inferred Phylogeny

We evaluated our DCNs-alignment-based phylogeny inference method on six different combinations of 398 single-chromosome prokaryotic species (strains). We benchmarked our DCNs-based method and compared its performances with that of five other state-of-the-art phylogeny inference methods (**Table 13**). These methods include (1) BPhyOG [46], a method based on overlapping genes (OG); (2) a method based on Composition Vector Tree (CVT) with *k* = 5, where *k* is the length of strings [47]. The concept of “Composition Vector Tree” which used the string appearance frequencies to represent a genome, and the distance between two genomes was calculated as the Euclidean distance between the two “Composition Vector Trees”. (3) [48] extended (2) by using the appearance frequencies of all the strings with length *k*< = 5, and named this vector “Complete Composition Vector”. (4) A method based on Structural Protein Domain Universe Graph (PDUG) [49]. This method incorporates a graph consisted of protein domains, which, to some extent, is similar to our DCNs-based method, but these two methods still have big differences. PDUG consists of all the protein domains and may be derived from several species, with known protein structures. Each of these domains is treated as a node in the PDUG. The protein structure and the structural similarity are based on the structural classification of protein “fold”, and the structural similarity is used to define the edge between two nodes. An organism is assigned nodes from the PDUG based on sequential similarities, and the distance matrix between graphs is generated based on the degree distribution. (5) ComPhy [50], a method based on gene Composite Distance (CD). Gene composite distance combines Gene Dispersion Distance, Genome Breakpoint Distance, and Gene Content Distance, and it achieves higher than 90% accuracy on all the six datasets. Detailed descriptions about ComPhy can be found at [50].

**Table 13:** Accuracy comparisons between our DCNs-based method and other methods for phylogeny inference.

Dataset	Species Num.	OG^1^	CVT (k = 5)^2^	CCV (k< = 5)^3^	PDUG^4^	CD^5^	DCNs
1	52	83.93	88.29	87.82	N/A	90.29	85.71
2	53	85.49	87.92	86.27	N/A	90.74	87.00
3	398	85.52	78.86	79.03	N/A	90.07	76.71
4	181	80.34	87.19	87.19	N/A	98.30	82.75
5	277	81.89	83.19	83.28	N/A	90.71	80.42
6	54	88.27	91.47	91.39	81.57	96.55	93.45

The accuracy of OG, CVT, SDD, and CD are directly retrieved from [50]. Accuracies are reflected by the percentage of the agreed quartets.

1 OG = Overlapping Gene Distance [46],

2 CVT = Composition Vector Tree [47], and *k* is the length of string,

3 CCV = Complete Composition Vector [48],

4 PDUG = Structural Protein Domain Universe Graph [49],

5 CD = Composite Distance [50].

**Figure 6:**
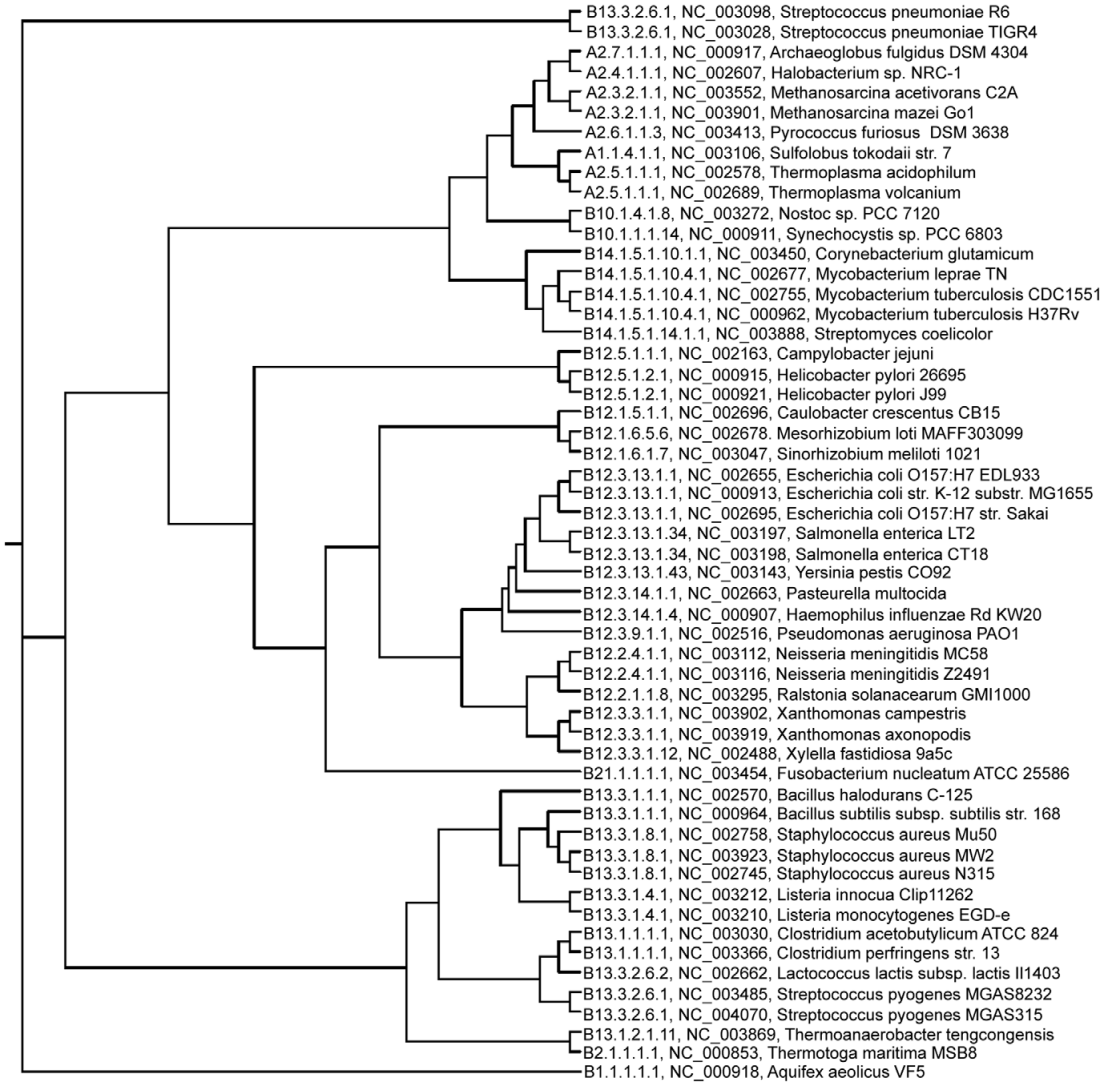
The phylogenetic tree generated on 54 single-chromosome prokaryotic taxa by our DCNs-alignment-based inference method. For each organism, the Bergey's code, NCBI ID, and the scientific name are shown. The percentage of the agreed quartets between this tree and the Bergey's taxonomy is 93.45%.

**Table 13** reports the results of our novel DCNs-based method on the six datasets. It performs very well on dataset 6, with similarity score 93.45% to Bergey's taxonomy, which contains 54 genomes that cover almost all the major clusters of the 398 genomes (**Table 14**). The phylogenetic tree generated on dataset 6 is shown in **Figure 6**, which largely complies with Bergey's taxonomy. Our experiments also show that DCNs-based method is robust as it has >85% accuracy on both datasets 1 and 2 which contain randomly selected species. Dataset 2 contains 52 species randomly selected from 398 chromosomes, whereas dataset 2 contains 53 species, half of which are randomly selected from Archaea and half randomly selected from Eubacteria. The accuracies on Datasets 4 and 5 are 82.75% and 80.42%, respectively, which shows DCN-based method has a decent sensitivity to distinguish and classify closely-related species, i.e. in the deeper level of the phylogenetic tree, as dataset 4 contains only genomes from Bacterial Division 12, and dataset 5 contains both Division 12 and 13. The overall accuracy on all the 398 chromosomes is 76%, which is slightly lower but still comparable with most of the other methods. In general, our DCNs-based method gains a comparable performance when compared to most of the other methods. ComPhy consistently showed a higher than 90% agreed percentage with Bergey's taxonomy. However, our DCNs-based method is based on organism-specific DCNs and graph alignment algorithm, which are completely different to the gene-based and sequence-based methods. It is likely that combining our DCNs-based method with the method in ComPhy can further improving the performance of ComPhy. Moreover, once the DCNs are constructed for candidate species, the graph alignment process is very fast and memory-efficient, because it does not need to consider all the genes in the whole chromosome, but only the nodes of DCNs, and on average one DCN of the 398 single-chromosome organisms contains 377 vertices. Using our PERL implementation of our graph alignment, with 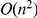 complexity (*n* is the number of unique domains in the two DCNs), the average time of aligning two DCNs of the 398 species is ∼1.6 seconds using a single 2.4 GHz Intel(R) Xeon CPU at a 64-bit Linux machine.

**Table 14:** The composition of dataset 6 containing 54 single-chromosome prokaryotic organisms.

Division	Bergey's code	Number of species
Bacteria *Aquificae*	B1	1
Bacteria *Fusobacteria*	B21	1
Bacteria *Thermotogae*	B20	1
Bacteria *Cyanobacteria*	B10	2
Bacteria *Actinobacteria*	B14	5
Bacteria *Firmicutes*	B13	15
Bacteria *Proteobacteria*	B12	21
Archaea	A2	8

### Conclusions and Future Work

We present a set of new DCN-based methods to predict protein function and to infer species phylogeny. We tested our methods on the genomes of several representative species and a large phylogeny benchmark. The results showed that DCN-based methods can predict protein function rather accurately, probably because DCNs constructed from all the proteins of a genome are rather complete and reliable in comparison with other protein networks. Our unique approach of constructing phylogeny by aligning species' DCNs also yields good performance that are comparable to other established methods, making it a complementary and valuable addition to the repository of phylogenetic analysis tools.

Despite initial promising results, there is still room to improve DCN-based function prediction and phylogeny inference. The “domains” used in our current version of DCNs are “domain families” defined by the Pfam database, which sometime could be further divided into sub-units. Using a more specific definition of domains might show different characteristics of DCNs. For example, we plan to use the structural domain definitions in the SCOP database [51] and ProDom [32] in our next study. However, since probably none of the current domain definitions is perfect and there is no unified ways of defining a domain, some noises are not completely avoidable.

In our current work, when predicting the GO terms of a target domain, we treated different GO terms in the neighboring domains independently. However, the semantic similarities between GO terms may be incorporated into the SVM and neighbor counting methods to improve protein function prediction. More advanced graph-kernels and random-walk graph kernels [52]–[54] can also be applied to DCNs to predict domain functions in order to take non-immediate neighboring domains into account. Similarly, advanced graph alignment algorithms, such as IsoRankN [55] may be used to align DCNs for phylogeny inference. Furthermore, the quality of the genome assembly and annotation is important to our DCNs-based phylogeny inference method. It may be interesting to find how annotation quality influences the performance of DCN-based phylogeny inference method by using different versions of annotated genomes.

## Materials and Methods

### Construction of Domain Co-occurrence Networks

The only input data needed for constructing a domain co-occurrence network (DCN) is the whole genome protein sequences of the target organism. In order to have a broad coverage of various species with known genome sequences and cover several model species for our experiments, we construct DCNs for *Homo sapiens* (human, downloaded from NCBI: human genome resources ftp://ftp.ncbi.nih.gov/genomes/H_sapiens/), *Saccharomyces cerevisiae* (yeast, downloaded from SGD [56]), *Caenorhabditis elegans* (downloaded from http://www.uniprot.org/uniprot/?query=organism:6239keyword:181), *Drosophila melanogaster* (fruit fly, downloaded from http://www.uniprot.org/uniprot/?query=organism:7227keyword:181), 15 plants species, and 398 single-chromosome prokaryotic species (downloaded from NCBI: microbial complete genomes taxonomy ftp://ftp.ncbi.nih.gov/genomes/Bacteria/). The 15 plant species include: *Chlamydomonas reinhardtii* (green alga), *Ostreococcus lucimarinus*, *Ostreococcus tauri*, *Ostreococcus RCC809*, *Chlorella vulgaris*, *Volvox carteri*, *Physcomitrella patens* (moss), *Selaginella moellendorffii* (gemmiferous spikemoss), *Oryza sativa* (rice), *Zea mays* (maize), *Sorghum bicolor* (sorghum), *Vitis vinifera* (grape), *Arabidopsis thaliana*, *Populus trichocarpa* (black cottonwood), and *Glycine max* (soybean). The protein sequences of *Arabidopsis thaliana* were downloaded from TAIR8 [57] (version 8), *Oryza sativa* (rice) from the “TIGR rice genome annotation resource” [58], *Zea mays* (maize) from the “MAGI website” (http://magi.plantgenomics.iastate.edu/), *Vitis vinifera* (grape) from “Genoscope” (http://www.genoscope.cns.fr/externe/GenomeBrowser/Vitis/), *Glycine max* (soybean) from “phytozome” website (http://www.phytozome.net/soybean), and the others from the Joint Genome Institute (JGI) website (http://www.jgi.doe.gov/). These 15 plants species represent virtually all major evolutionary stages of plants, including alga, primitive land plants, and higher plants.

**Figure 7:**
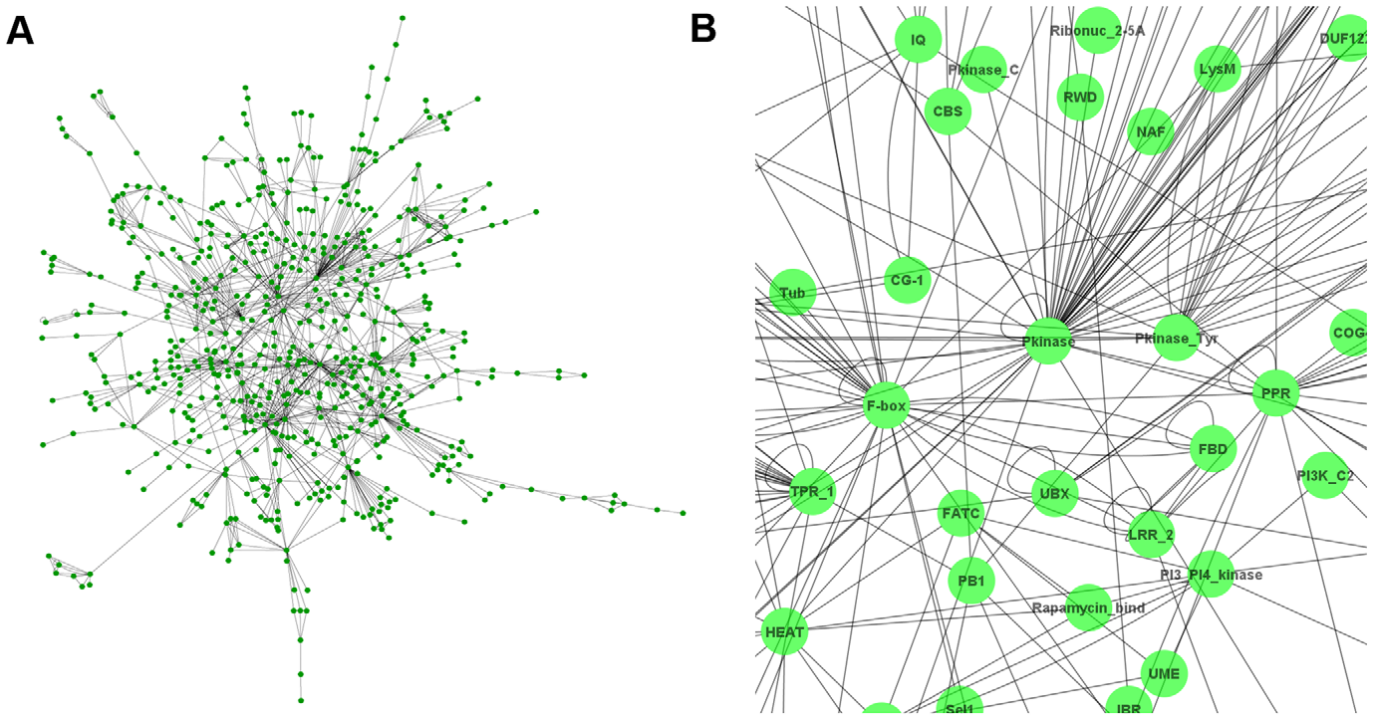
Domain co-occurrence network of Arabidopsis thaliana. In total, the DCN of Aradbisopsis contains 141 disconnected sub-graphs (each one has no edges connecting to any other sub-graphs); and most of the sub-graphs have less than 10 nodes. (A) is the largest DCN sub-graph, or the main graph, of Arabidopsis DCNs that has 626 nodes and 1,304 edges. The graphs shown in **Figure 1** and **8** are two acutal examples of the small sub-graphs in Arabidopsis DCN. (B) is an enlarged partial view of Arabidopsis main DCN, in which domain *Pkinase* is a hub.

The program PfamScan is downloaded from the Pfam [33] FTP site (ftp://ftp.sanger.ac.uk/pub/databases/Pfam/Tools/), together with Pfam databases and hidden Markov model (HMM) libraries. This domain searching tool is locally installed, which incorporates HMMER (http://hmmer.janelia.org/) and BLAST to search against Pfam domain libraries. Each protein sequence of the target species was searched against Pfam using PfamScan. The domain hits with an e-value< = 0.01 were kept. When constructing a DCN, each domain is considered as a node, or vertices, in the undirected graph; and every two domains (i.e. nodes) are connected by an edge if they co-exist in one protein [26] as shown in **Figure 1**. **Figure 7** illustrates the main DCN of *Arabidopsis thaliana* proteome visualized by Cytoscape [59].

### Domain function prediction – GO terms

**Figure 8:**
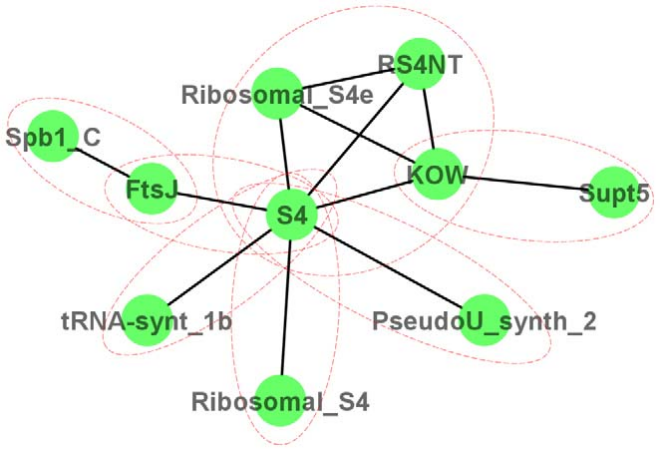
The relationship between sub-graphs and protein function. Each red eclipse encircles the domains co-occurred in one protein.

Because domains involved in the same biological process are more likely to co-occur in one protein, domains with similar functions tend to cluster in DCNs. **Figure 8** shows a densely connected sub-graph identified in the DCN of Arabidopsis. This sub-graph consists of 10 domains, which form seven different proteins in Arabidopsis. The domains of each protein are circled by red dotted-line eclipse. Not surprisingly, all the proteins are identified to participate in RNA synthesis processes according to the Pfam annotations. Thus, the function of one protein (e.g. *spb1_C*+*FtsJ*) can be inferred from another protein (e.g. *KOW*+*Supt5*) that is connected through a path in the sub-graph and the central node, e.g. domain *S4*.

In order to demonstrate the potential of DCNs to infer protein function, we first focused on predicting the specific GO terms [60] of a domain from its neighboring nodes. Taking **Figure 8** as an example, if we pretend to not know the GO terms of domain *S4*, we can use the GO terms of its radius one (immediate) neighboring domains: *FtsJ*, *tRNA-synt_1b*, *Ribosomal_S4*, *PseudoU_synth_2*, *KOW*, and *Ribosomal_S4e* to infer the GO terms of the central domain. We can also do that by incorporating radius two neighboring domains, including *Spb1_C*, *Supt5*, and *RS4NT*. In our experiments of predicting domain functions, we used *Arabidopsis thaliana*, *Saccharomyces cerevisiae*, and *Homo sapiens* as benchmarks. We included *Arabidopsis thaliana* because it has been well-studied as a model plant species and has high-quality genome annotation. We incorporated *Saccharomyces cerevisiae* and *Homo sapiens* because they are often used as model species for protein function prediction.

Three methods were used in our experiments to predict domain functions. The first is the most straightforward method, majority vote or neighbor-counting: count the appearance number of every GO term occurred in the neighboring nodes of the target domain; and then rank the GO terms based on this occurrence frequency; and the top ranked GO term(s) is (are) considered as the predicted GO term(s). This method was used by [61] to predict protein functions based on yeast protein-protein interaction networks.

The second method in our experiment considers the distribution of every GO term in the entire DCN. This method was used by [62] to improve the performances of the neighbor-counting method mentioned above. This method ranks GO terms gathered from the neighboring domains by a χ^2^ value:
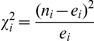
where *i* denotes a GO term, for example, GO:0090295, *negative regulation of transcription by nitrogen catabolites*; *n_i_* denotes the observed number of GO term *i* in *m*-neighboring domains; and *e_i_* denotes the expected number of GO term *i* appeared in *m* nodes.

Besides the above two methods, we also designed a Support Vector Machine (SVM) -based method. For each domain, we generated a feature-vector which included: (1) The occurrence frequency of each of the 31,398 GO terms defined by Gene Ontology [60], gathered from the neighboring nodes of the target domain. For example, if there are in total two GO terms occurring in the neighboring nodes, and each of them occurs once, the frequencies of both of these two GO terms are 0.5, and the frequencies of all the other GO terms are 0. (2) The occurrence frequency of each of the six enzyme families collected from the neighboring nodes of the target domain. (3) The occurrence frequency of each of the 20 amino acids of the target domain. From all the proteins of the target species, the ones that are found to have the target domain are gathered; and then the segments of the domain region are used to calculate the occurrence frequency of the 20 amino acids. (4) Secondary structure information: the occurrence frequency of helix (H), strand (E), and coil (C) of the target domain, which are calculated in a similar way as in (3). (5) Solvent accessibility information: the occurrence frequency of solvent exposed and buried amino acids of the target domain. The secondary structures and solvent accessibilities are predicted by SCRATCH [63]. Some of these features, such as secondary structure, amino acid sequence, and solvent accessibilities, have been widely used in protein function prediction [43], [64]–[66]; therefore, it seemed reasonable to also test them in DCN-based predictions. However, our experiments showed that not all of these features made positive contributions to improvements in accuracy. Details are discussed in the “**Results and Discussion**” section.

**Figure 9:**
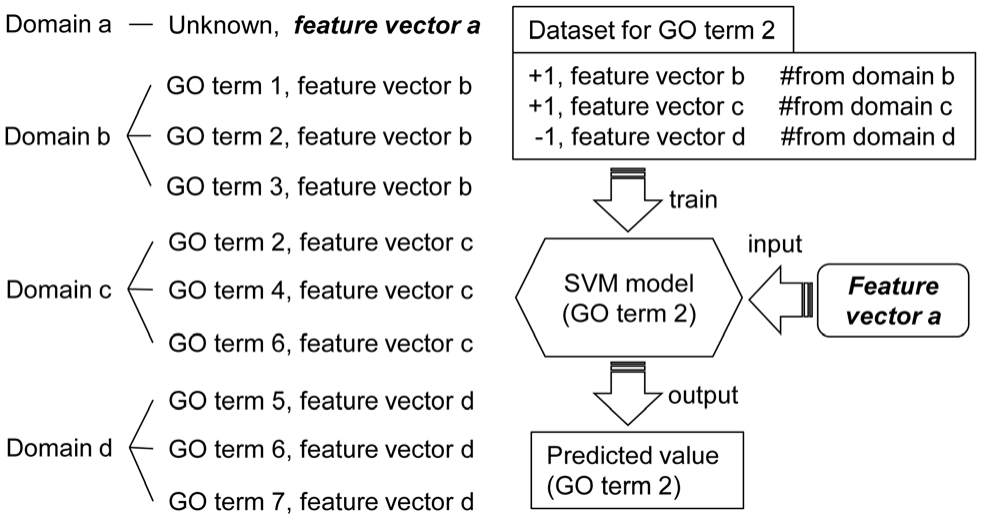
Predicting the GO terms of domain *a* using SVM classification. The DCN is supposed to have only four domains: *a*, *b*, *c*, and *d*, and the functions of domain *a* are treated unknown. It only shows the binary classification process for GO term *2*. This same process should be applied on every GO term (GO term *1*–*7*) that occurred in the training domains (domain *b*, *c*, and *d*). The top ranked GO term(s) is (are) treated as the final predicted functions.

According to TAIR8, Arabidopsis has 1,454 domains in total. 736 of these domains have at least one GO term available in Pfam (i.e. domains with known function) and have at least one GO term available in its radius one neighboring nodes. From these 736 nodes, we performed a leave-one-domain-out cross-validation. **Figure 9** shows an example, in which we supposed there are in total only four domains existing in the DCN. Each time we left one domain out, which is domain *a* in **Figure 9**, and treated its GO terms unknown. From the remaining domains, which are domain *b*, *c*, and *d* in **Figure 9**, we generated a feature vector from each of these domains. A training dataset for each GO term was then constructed in which the domains having the function of this GO term were labeled positive and the ones not were labeled as negative examples. **Figure 9** shows the training dataset for “GO term 2”. If one domain contains several GO terms, which happens quite often, each of the GO terms is included as positive examples in its training dataset. As shown in **Figure 9**, “GO term 2” occurs in both domains *b* and *c*, so the training dataset of “GO term 2” contains two positive examples with feature vector *b* and *c*. The negative examples are randomly selected from all of the domains that do not have the GO term. If a feature vector of a domain has been included as a positive example, it will not be selected as a negative example.

A binary SVM model was trained for each of the GO terms occurring in the remaining domains using SVM*^light^* [67], and then the target domain, domain *a* in **Figure 9**, was classified by each of these SVM models. Every SVM model generates a predicted value, based on which all the GO terms are ranked, and the top ranked GO terms are considered as the predicted GO terms. In Arabidopsis, 467 models on average were trained every time when classifying a target domain, and 403 for yeast, and 631 for human.

Many nodes in the DCN have a self-loop, i.e. an edge starting from and ending to the same node. If a domain with self-loop is selected as a target domain, it also exists in its own radius one neighboring domains, which makes the GO terms of the neighboring domains contain the real GO term(s) of the target domain. Therefore, the self-loop of every target domain, if exists, is removed.

### Domain function prediction – enzyme family

We also used DCN to predict whether a domain is an enzyme domain; and if so, which of the six Enzyme Commission (EC) classes it belongs to. These six enzyme classes are: *Oxidoreductases*, *Transferases*, *Hydrolases*, *Lyases*, *Isomerases*, and *Ligases*, which have an EC number starting from 1 to 6, respectively. A mapping file between GO terms and Enzyme Commission (EC) numbers was downloaded from Gene Ontology website (http://www.geneontology.org/external2go/ec2go). If a domain contains at least one GO term that maps to an EC number, this domain is considered as an enzyme domain.

To predict whether a domain is an enzyme domain, we applied SVM along with all the features mentioned in the previous section. In Arabidopsis, there are a total of 307 enzyme domains that have at least one GO term or EC number available/known in the radius one neighboring domains, i.e. the GO term and EC classes occurrence frequency are not all 0. Each of these domains was considered as a positive example. The negative examples consisted of the domains with known functions that are not enzyme-related, i.e. none of its GO terms maps to an EC number. In this way, we eliminated the “Domain with Unknown Function” (DUF) domains, because these domains may not be non-enzyme domains. We gathered a total of 429 negative domains/examples in Arabidopsis. A binary SVM model was built by SVM*^light^* [67], and different kernels and combinations of features tested by several leave-one-out cross-validations. Besides SVM, we also tested a neighbor-inference method, by which if the neighboring nodes contain at least one EC number, we predicted the target (central) domain an enzyme domain; otherwise, non-enzyme domain.

Each of the 307 Arabidopsis enzyme domains having an enzyme class number starting from 1 to 6 based on the first digit of their EC numbers. SVM*^multi-class^* [67] was used to build SVM models and classify a query domain into one of the six classes. A leave-one-out cross-validation was performed on these 307 enzyme domains, which contain 90 domains in EC class 1, 91 domains in EC class 2, 59 domains in EC class 3, 19 domains in EC class 4, 13 domains in EC class 5, and 37 domains in EC class 6.

### Protein function prediction

To predict the functions of a query protein, we used a DCN-based aggregated neighbor-counting method. Given the amino acid sequence of a query protein, we run a profile-profile alignment tool HHsearch [44] against Pfam profile database (downloaded at ftp://toolkit.lmb.uni-muenchen.de/HHsearch/databases/) to detect Pfam domains. In order to determine the most relevant species of the query proteins, a PSI-BLAST search was performed against the whole genome protein sequences of *H. sapiens*, *S. cerevisiae*, *C. elegans*, *D. melanogaster*, 15 plants species, and 398 single-chromosome prokaryotic species, and the species having the most significant PSI-BLAST hit (with the minimum e-value) was considered the relevant species. The DCN of the relevant species was used to make functional prediction for the query protein. The aggregated neighbor-counting was then used to predict functions. From the HHsearch result report of each query protein, the detected Pfam domain(s) with an e-value< = 0.01 was (were) extracted. For each of these extracted domain(s), we retrieved its (their) radius one neighboring domains, and the neighboring domains of all extracted domains were put together. The GO terms for these neighbor domains were retrieved from Pfam database and ranked by their occurrence frequencies. This list of ranked GO terms was our final prediction. **Figure 4** shows an example of using the aggregated neighbor-counting method to predict protein functions.

### Phylogenetic tree construction and its evaluation

To construct a phylogenetic tree of a group of species, we aligned their DCNs to identify conserved sub-networks, or common network topology, which can reveal the evolutionary significant patterns between species. This novel method is different from existent sequence-based methods, as when comparing the network topologies, since it uses the entire proteome of each species to infer the phylogenetic relationship.

To align the DCNs of two species *a* and *b*, we define the DCN of species *a* as a graph 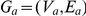, where 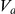 is the set containing all the vertices of graph 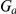, and 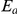 is the set containing all the non-redundant edges in graph 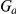. Similarly, we define the DCN of species *b* as a graph 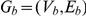. The mutual vertices between graph 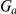 and graph 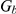 (i.e., the same domains exist in both the DCNs of species *a* and *b*) are defined as set 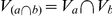. We identified the mutual vertices 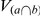, and calculate NUM(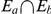) - the number of mutual edges between graph 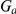 and graph 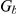. Because we do not assign weights to edges, two edges from two graphs connecting the same vertices are considered equal. Then we count the total number of unique edges connecting only mutual vertices in both graph 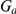 and graph 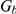, denoted as NUM(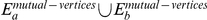). Then the similarity score 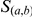 between graph 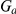 and graph 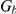 is calculated as the number of mutual edges, divided by the total number of unique edges on the mutual vertices:
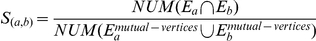

**Figure 5** illustrates an example of the graph alignment algorithm, in which we align the two graphs as **Figure 5 **(**A**) and (**B**). We at first find the mutual vertices between (**A**) and (**B**), which are *b*, *c*, *d*, and *e* as shown in **Figure 5 **(**C**). Then three mutual edges between graph (**A**) and graph (**B**): *c*–*d*, *c*–*e*, and *e*–*d* are picked as shown in **Figure 5 **(**D**). The seven unique edges on the mutual vertices between graph (**A**) and (**B**) are shown in **Figure 5 **(**C**). In this case, there are two edges occurred between node *c* and *d*, which is due to the fact that two proteins contain both domain *c* and *d*. These two redundant edges are considered as one edge. Thus the number of unique edges on the common vertices is 6. Therefore, the similarity score between graph **Figure 5 **(**A**) and (**B**) is equal to the number of edges in graph **Figure 5 **(**D**) divided by the number of unique edges in graph **Figure 5 **(**C**), which is 3/6 = 0.5.

This alignment algorithm is straightforward, easy to implement, and has low computational complexity compared to complicated global alignment algorithms. If *n* is the total number of unique domains in the two DCNs, the computational complexity will be at most 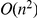. In the future, we plan to try more advanced global graph-alignment algorithms, such as IsoRankN [55].

Given a group of species, we used our graph alignment algorithm for pair-wise comparisons and generated a distance matrix (distance score = 1 - similarity score). We generated a phylogenetic tree using the program “NEIGHBOR” in the phylogeny inference package PHYLIP [68], which implements the neighbor joining method [69].

Unlike the phylogenetic study of more advanced organisms, which has plenty of morphology and archeology evidence available, there are still uncertainties in bacteria taxonomy. However, the scientific community usually considers the classification presented in the book *Bergey's Manual of Determinative Bacteriology* [70] as the best approximation. Bergey defines a set of taxonomy codes to indicate the classification. For example, the organism *Lactobacillus casei* has a Bergy's code B13.3.2.1.1 indicating it belongs to kingdom *Bacteria*, Division 13 (*Firmicutes*), Class 3 (*Bacilli*), Order 2 (*Lactobacillales*), Family 1 (*Lactobacillaceae*), and Genus 1 (*Lactobacillus*). In our work, we use Bergey's classification as the reference, and compared our phylogenetic trees to this reference. The similarity between our phylogenetic tree and Bergey's classification was calculated by ComPhy [50], which counts the number of agreed quartets between our phylogenetic tree and Bergey's classification, and uses the percentage of the agreed quartets as the similarity measure, whereas a quartet is a sub-graph topology containing four taxa (tree nodes) [50].

**Figure 10:**
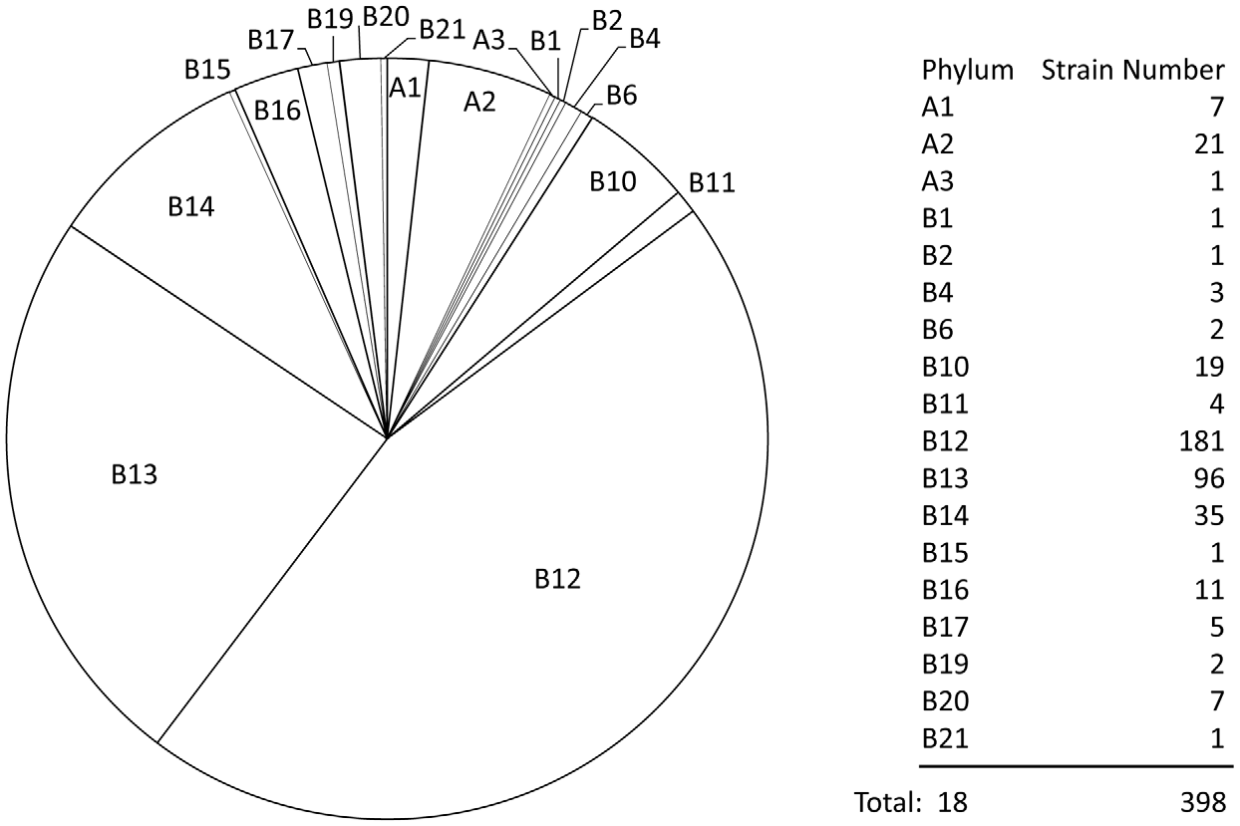
Composition details of the 398 single-chromosome prokaryotic genomes (strains).

In order to comprehensively evaluate the potential of DCNs to infer phylogeny, we conducted experiments on six datasets with different combinations of 398 single-chromosome prokaryotic genomes (strains). These six datasets were previously used to compare several different phylogeny inference methods [50]. For direct comparison, we used the same version of the datasets: 432 prokaryotic genomes downloaded from NCBI in September 2007. After removing 34 multi-chromosome species, we included 398 species in our dataset, which contains 29 Archaea species and 369 Eubacteria species (**Figure 10**). Dataset 1 consists of 52 randomly selected species from Bergey's taxonomy tree [50]. Dataset 2 contains 53 species, 28 of which are randomly selected from the Archaea species, and 25 of them are randomly selected form Eubacteria species. Dataset 3 contains all the 398 organisms. Dataset 4 is composed by Bacterial Division 12 (181 species). Division 12 is a large division containing approximately half of the 398 genomes. Dataset 5 is formed by Bacterial Division 12 (181 species) and Division 13 (96 species). These two are big clusters, and the phylogenetic tree generated on dataset 5 should contain two tiger clusters. Dataset 6 contains 54 organisms, which were obtained from Deeds [49], a phylogeny inference method using domain structures networks.

## Supporting Information

Figure S1 **The node degree distributions of** ***H. sapiens*** **,** ***S. cerevisiae*** **,** ***C. elegans*** **,** ***D. melanogaster*** **, and 15 plant genomes.**
(TIF)

Figure S2 **The shortest path length distributions of** ***H. sapiens*** **,** ***S. cerevisiae*** **,** ***C. elegans*** **,** ***D. melanogaster*** **, and 15 plant genomes.**
(TIF)

Figure S3 **The average clustering coefficient distributions of** ***H. sapiens*** **,** ***S. cerevisiae*** **,** ***C. elegans*** **,** ***D. melanogaster*** **, and 15 plant genomes.**
(TIF)

## References

[1] Hartwell L, Hopfield J, Leibler S, Murray A (1999). From molecular to modular cell biology.. Nature.

[2] Ideker T, Galitski T, Hood L (2001). A New Approach To Decoding Life: Systems Biology.. Annual Review of Genomics and Human Genetics.

[3] Kitano H (2002). Computational systems biology.. Nature.

[4] Hucka M, Finney A, Sauro H, Bolouri H, Doyle J (2003). The systems biology markup language (SBML): a medium for representation and exchange of biochemical network models.. Bioinformatics.

[5] Cheng J, Scharenbroich L, Baldi P, Mjolsness E (2005). Sigmoid: towards an intelligent, scalable, software infrastructure for pathway bioinformatics and systems biology.. IEEE Intelligent Systems.

[6] Bonneau R (2008). Learning biological networks: from modules to dynamics.. Nature chemical biology.

[7] Zhang A (2009).

[8] Barabasi A, Oltvai Z (2004). Network biology: understanding the cell's functional organization.. Nature Reviews Genetics.

[9] Elowitz M, Leibler S (2000). A synthetic oscillatory network of transcriptional regulators.. Nature.

[10] Segre D, Vitkup D, Church G (2002). Analysis of optimality in natural and perturbed metabolic networks.. Proceedings of the National Academy of Sciences of the United States of America.

[11] Uetz P, Giot L, Cagney G, Mansfield T, Judson R (2000). A comprehensive analysis of protein-protein interactions in Saccharomyces cerevisiae.. Nature.

[12] Rinner O, Mueller L, Hubalek M, Muller M, Gstaiger M (2007). An integrated mass spectrometric and computational framework for the analysis of protein interaction networks.. Nature biotechnology.

[13] Singh R, Xu J, Berger B (2008). Global alignment of multiple protein interaction networks with application to functional orthology detection.. Proceedings of the National Academy of Sciences.

[14] Hakes L, Pinney J, Robertson D, Lovell S (2008). Protein-protein interaction networks and biology - what's the connection?. Nature biotechnology.

[15] Ramirez F, Schlicker A, Assenov Y, Lengauer T, Albrecht M (2007). Computational analysis of human protein interaction networks.. Proteomics.

[16] Lewis A, Jones N, Porter M, Charlotte D (2010). The function of communities in protein interaction networks at multiple scales.. BMC Systems Biology.

[17] Li F, Li P, Xu W, Peng Y, Bo X (2010). PerturbationAnalyzer: a tool for investigating the effects of concentration perturbation on protein interaction networks.. Bioinformatics.

[18] Agarwal S, Deane C, Porter M, Jones N (2010). Revisiting date and party hubs: Novel approaches to role assignment in protein interaction networks.. PLoS Comput Biol.

[19] Nguyen T, Jordan F (2010). A quantitative approach to study indirect effects among disease proteins in the human protein interaction network.. BMC Systems Biology.

[20] Wu G, Feng X, Stein L (2010). A human functional protein interaction network and its application to cancer data analysis.. Genome Biology.

[21] Ito T, Chiba T, Ozawa R, Yoshida M, Hattori M (2001). A comprehensive two-hybrid analysis to explore the yeast protein interactome.. Proc Natl Acad Sci.

[22] Scott J, Ideker T, Karp R, Sharan R (2006). Efficient algorithms for detecting signaling pathways in protein interaction networks.. Journal of Computational Biology.

[23] Chen X, Liu M, Ward R (2008). Protein function assignment through mining cross-species protein-protein interactions.. PLoS ONE.

[24] Zhang C, Joshi T, Lin G, Xu D (2009). An integrated probabilistic approach for gene function prediction using multiple sources of high-throughput data.. Int J of Computational Biology and Drug Design.

[25] Bork P, Jensen L, von Mering C, Ramani A, Lee I (2004). Protein interaction networks from yeast to human.. Current Opinion in Structural Biology.

[26] Wuchty S, Almaas E (2005). Evolutionary cores of domain co-occurrence networks.. BMC Evolutionary Biology.

[27] Wuchty S (2001). Scale-free behavior in protein domain networks.. Molecular biology and evolution.

[28] Fong J, Geer L, Panchenko A, Bryant S (2007). Modeling the evolution of protein domain architectures using maximum parsimony.. Journal of Molecular Biology.

[29] Sarah K, Sarah T (2009). Protein domain organisation: adding order.. BMC Bioinformatics.

[30] Ekman D, Bjirklund A, Frey-Skott J, Elofsson A (2005). Multi-domain proteins in the three kingdoms of life: orphan domains and other unassigned regions.. Journal of Molecular Biology.

[31] Sonnhammer E, Eddy S, Birney E, Bateman A, Durbin R (1998). Pfam: multiple sequence alignments and HMM-profiles of protein domains.. Nucleic Acids Research.

[32] Servant F, Bru C, Carrere S, Courcelle E, Gouzy J (2002). ProDom: automated clustering of homologous domains.. Briefings in Bioinformatics.

[33] Bateman A, Coin L, Durbin R, Finn R, Hollich V (2004). The Pfam protein families database.. Nucleic Acids Research.

[34] Watts D, Strogatz S (1998). Collective dynamics of ‘small-world’ networks.. Nature.

[35] Ravasz E, Somera A, Mongru D, Oltvai Z, Barabasi A (2002). Hierarchical organization of modularity in metabolic networks.. Science.

[36] Li D, Li J, Ouyang S, Wang J, Wu S (2006). Protein interaction networks of Saccharomyces cerevisiae, Caenorhabditis elegans and Drosophila melanogaster: large-scale organization and robustness.. Proteomics.

[37] Albert R, Jeong H, Barabasi A (2000). Error and attack tolerance of complex networks.. Nature.

[38] Jeong H, Tombor B, Albert R, Oltvai Z, Barabasi A (2000). The large-scale organization of metabolic networks.. Nature.

[39] Du Z, Li L, Chen C, Yu P, Wang J (2009). G-SESAME: web tools for GO-term-based gene similarity analysis and knowledge discovery.. Nucleic Acids Research.

[40] Saigo H, Hattori M, Kashima H, Tsuda K (2010). Reaction graph kernels predict EC numbers of unknown enzymatic reactions in plant secondary metabolism.. BMC Bioinformatics.

[41] Komurov K, White M, Ram P (2010). Use of Data-Biased Random Walks on Graphs for the Retrieval of Context-Specific Networks from Genomic Data.. PLoS Comput Biol.

[42] Basu M, Carmel L, Rogozin I, Koonin E (2008). Evolution of protein domain promiscuity in eukaryotes.. Genome Research.

[43] Cai C, Han L, Ji Z, Chen Y (2004). Enzyme family classification by support vector machines.. Proteins: Structure, Function, and Bioinformatics.

[44] Soding J, Biegert A, Lupas A (2005). The HHpred interactive server for protein homology detection and structure prediction.. Nucleic Acids Research.

[45] Altschul S, Madden T, Schaffer A, Zhang J, Zhang Z (1997). Gapped BLAST and PSI-BLAST: a new generation of protein database search programs.. Nucleic Acids Research.

[46] Luo Y, Fu C, Zhang D, Lin K (2007). BPhyOG: an interactive server for genome-wide inference of bacterial phylogenies based on overlapping genes.. BMC Bioinformatics.

[47] Gao L, Qi J, Sun J, Hao B (2007). Prokaryote phylogeny meets taxonomy: An exhaustive comparison of composition vector trees with systematic bacteriology.. Science in China Series C: Life Sciences.

[48] Wu X, Cai Z, Wan X, Hoang T, Goebel R (2007). Nucleotide composition string selection in HIV-1 subtyping using whole genomes.. Bioinformatics.

[49] Deeds E, Hennessey H, Shakhnovich E (2005). Prokaryotic phylogenies inferred from protein structural domains.. Genome Research.

[50] Lin G, Cai Z, Chakraborty S, Xu D (2009). ComPhy: prokaryotic composite distance phylogenies inferred from whole-genome gene sets.. BMC Bioinformatics.

[51] Murzin A, Brenner S, Hubbard T, Chothia C (1995). SCOP: a structural classification of proteins database for the investigation of sequences and structures.. Journal of Molecular Biology.

[52] Borgwardt K, Ong C, Schonauer S, Vishwanathan S, Smola A (2005). Protein function prediction via graph kernels.. Bioinformatics.

[53] Lanckriet G, Deng M, Cristianini N, Jordan M, Noble W (2004). Kernel-based data fusion and its application to protein function prediction in yeast.. Pacific Symposium on Biocomputing.

[54] Hiroto S, Masahiro H, Hisashi K, Koji T (2010). Reaction graph kernels predict EC numbers of unknown enzymatic reactions in plant secondary metabolism.. BMC Bioinformatics.

[55] Liao C, Lu K, Baym M, Singh R, Berger B (2009). IsoRankN: spectral methods for global alignment of multiple protein networks.. Bioinformatics.

[56] Issel-Tarver L, Christie K, Dolinski K, Andrada R, Balakrishnan R (2002). Saccharomyces genome database.. Methods in enzymology.

[57] Rhee S, Beavis W, Berardini T, Chen G, Dixon D (2003). The Arabidopsis Information Resource (TAIR): a model organism database providing a centralized, curated gateway to Arabidopsis biology, research materials and community.. Nucleic Acids Research.

[58] Ouyang S, Zhu W, Hamilton J, Lin H, Campbell M (2006). The TIGR rice genome annotation resource: improvements and new features.. Nucleic Acids Research.

[59] Shannon P, Markiel A, Ozier O, Baliga N, Wang J (2003). Cytoscape: a software environment for integrated models of biomolecular interaction networks.. Genome Research.

[60] Ashburner M, Ball C, Blake J, Botstein D, Butler H (2000). Gene ontology: tool for the unification of biology.. Nature Genetics.

[61] Schwikowski B, Uetz P, Fields S (2000). A network of protein-protein interactions in yeast.. Nature biotechnology.

[62] Hishigaki H, Nakai K, Ono T, Tanigami A, Takagi T (2001). Assessment of prediction accuracy of protein function from protein-protein interaction data.. Yeast.

[63] Cheng J, Randall A, Sweredoski M, Baldi P (2005). SCRATCH: a protein structure and structural feature prediction server.. Nucleic Acids Research.

[64] Jensen L, Gupta R, Staerfeldt H, Brunak S (2003). Prediction of human protein function according to Gene Ontology categories.. Bioinformatics.

[65] Whisstock J, Lesk A (2004). Prediction of protein function from protein sequence and structure.. Quarterly reviews of biophysics.

[66] Borgwardt K, Kriegel H (2005). Kernel Methods for Protein Function Prediction..

[67] Joachims T, Schölkopf B, Burges C, Smola A, Advances in Kernel Methods - Support Vector Learning (1999). Making large scale SVM learning practical..

[68] Felsenstein J (1989). PHYLIP-phylogeny inference package (version 3.2).. Cladistics.

[69] Saitou N, Nei M (1987). The neighbor-joining method: a new method for reconstructing phylogenetic trees.. Molecular biology and evolution.

[70] Bergey D, Holt J (1994).

