# Supplementary figures and images for: A Protein Domain Co-Occurrence Network Approach for Predicting Protein Function and Inferring Species Phylogeny
Source: PLoS One. 2011 Mar 24;6(3):e17906. doi: 10.1371/journal.pone.0017906 (PMC3063783; doi:10.1371/journal.pone.0017906)

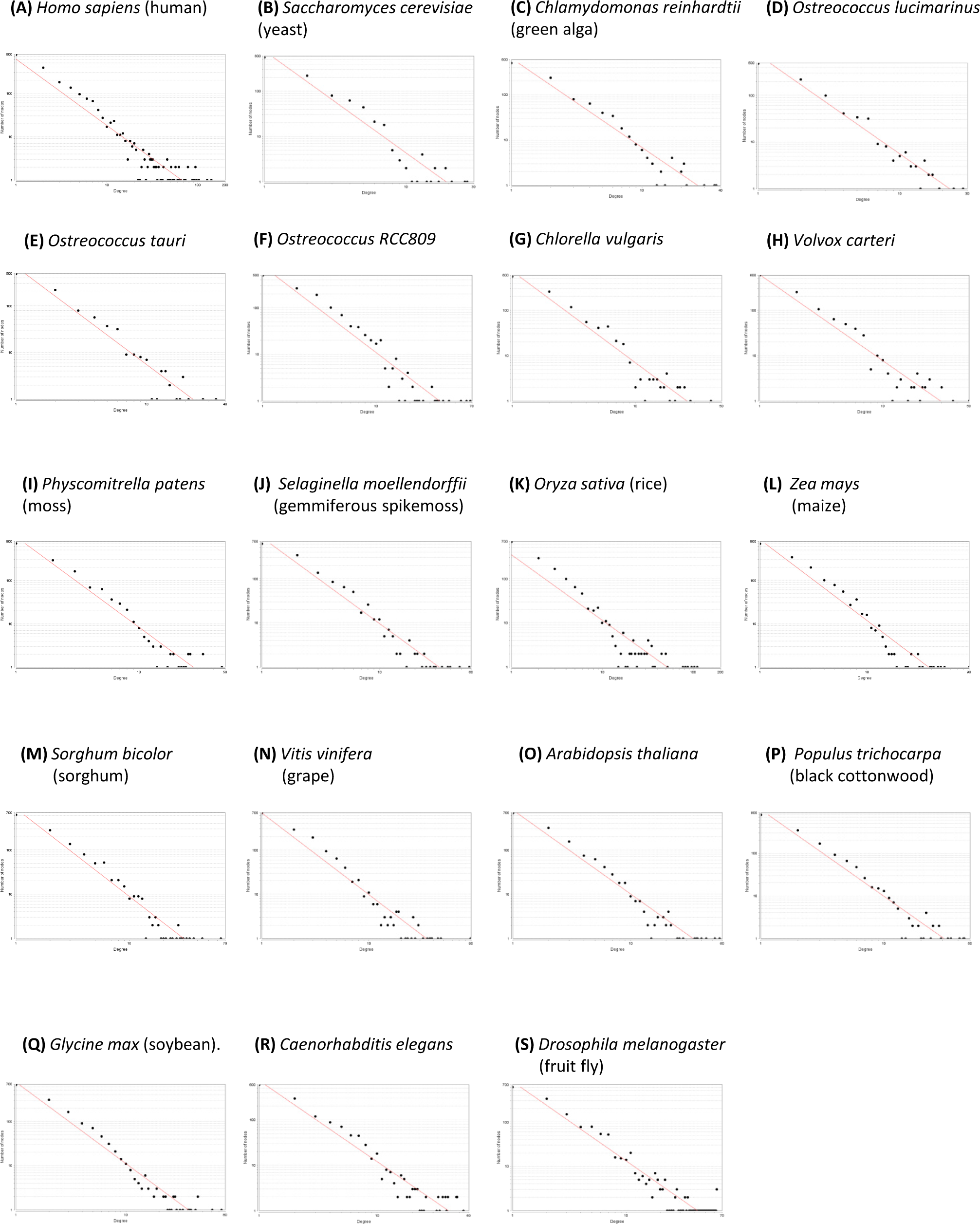

Supplement: Figure S1 — The node degree distributions of H. sapiens , S. cerevisiae , C. elegans , D. melanogaster , and 15 plant genomes. (TIF) [file pone.0017906.s001.tif]

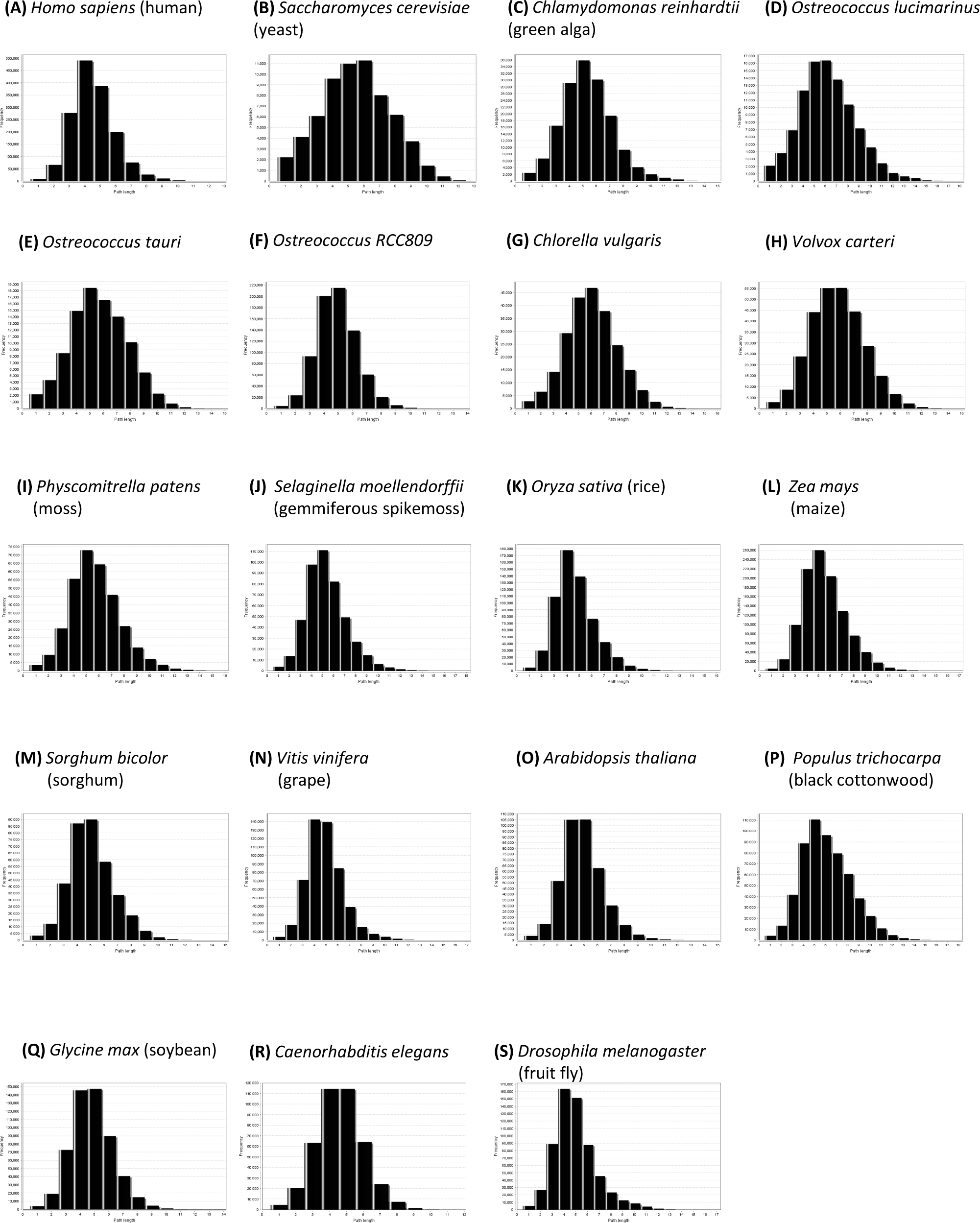

Supplement: Figure S2 — The shortest path length distributions of H. sapiens , S. cerevisiae , C. elegans , D. melanogaster , and 15 plant genomes. (TIF) [file pone.0017906.s002.tif]

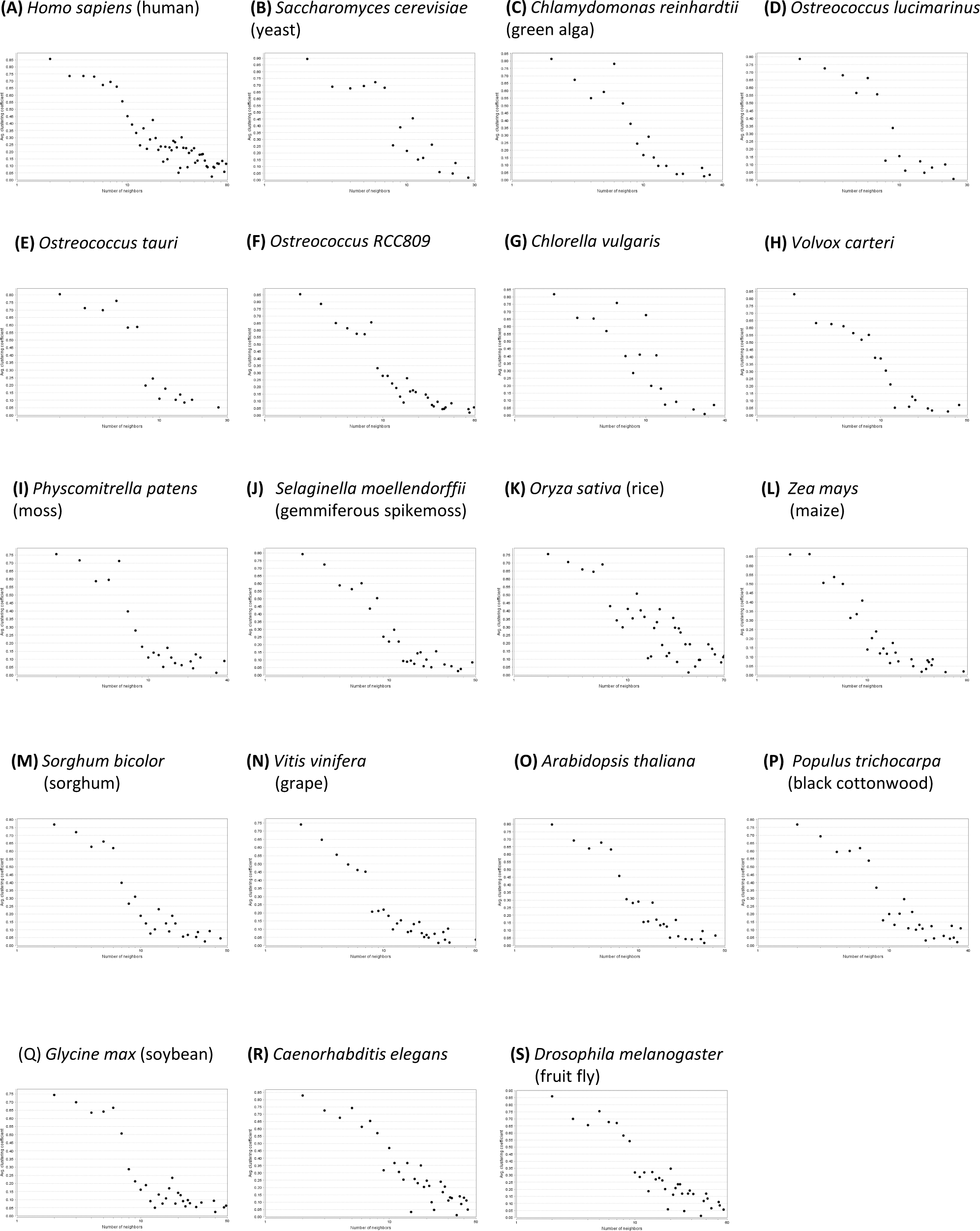

Supplement: Figure S3 — The average clustering coefficient distributions of H. sapiens , S. cerevisiae , C. elegans , D. melanogaster , and 15 plant genomes. (TIF) [file pone.0017906.s003.tif]
